# Unraveling the role of shrimp hydrolysate as a food supplement in the immune function and fecal microbiota of beagle dogs

**DOI:** 10.1038/s41598-025-09942-8

**Published:** 2025-07-15

**Authors:** Joana Guilherme-Fernandes, Carolina Barroso, Alexandra Correia, Tiago Aires, Timur Yergaliyev, Amélia Camarinha-Silva, Manuel Vilanova, António J. M. Fonseca, Sofia A. C. Lima, Margarida R. G. Maia, Ana R. J. Cabrita

**Affiliations:** 1 REQUIMTE, LAQV, ICBAS, School of Medicine and Biomedical Sciences, University of Porto, Porto, Portugal; 2 i3S, Instituto de Investigação e Inovação em Saúde, Universidade do Porto, Porto, Portugal; 3 ICBAS, School of Medicine and Biomedical Sciences, University of Porto, Porto, Portugal; 4 SORGAL, Sociedade de Óleos e Rações S.A., S. João Ovar, Portugal; 5 Hohenheim Center for Livestock Microbiome Research, HoLMiR, University of Hohenheim, Stuttgart, Germany; 6 Institute of Animal Science, University of Hohenheim, Stuttgart, Germany; 7 LEAF - Linking Landscape, Environment, Agriculture and Food Research Center, Associated Laboratory TERRA, Instituto Superior de Agronomia, Universidade de Lisboa, Lisboa, Portugal

**Keywords:** Immunology, Microbiology

## Abstract

The inclusion of protein hydrolysates from animal by-products in petfood may enhance the industry’s sustainability, while contributing to dogs’ health due to their nutritional and functional properties. However, inconsistent results of protein hydrolysates on gut microbiota, immune responses, among other health parameters, have been observed in dogs. This study aimed to evaluate the effects of diets supplemented with 5% shrimp hydrolysate, compared to non-supplemented diets, on hematological parameters, serum chemistry profile, innate and adaptive immune function, and fecal microbiota composition in adult Beagle dogs during a 12-week feeding trial. Dietary inclusion of shrimp hydrolysate decreased blood eosinophils (*P* = 0.017) and glucose levels (*P* = 0.023), while increasing levels of white blood cells (*P* = 0.002), platelets (*P* = 0.038), neutrophils (*P* = 0.036), and CD4^+^ (*P* < 0.001) and CD8^+^ T cells (*P* < 0.001) single producers of tumor necrosis factor-alpha. It also led to a more extensive proliferation of CD4^+^ T cells in response to LipL32 antigen (*P* = 0.020) and to a decrease in superoxide production in stimulated cells (*P* = 0.002). Additionally, supplemented diets increased *Oscillospiraceae* (*P-adj* = 0.005) and Clostridia (*P-adj* = 0.017) abundance, and decreased such of *Sellimonas* (*P-adj* = 0.025). Overall, the results indicate that diet supplementation with shrimp hydrolysate at 5% modulates the immune response and fecal microbiota, highlighting its potential to be included in hypoallergenic and gastrointestinal diets, and in diets for diabetic dogs.

**Supplementary Information:**

The online version contains supplementary material available at 10.1038/s41598-025-09942-8.

## Introduction

Hydrolyzed protein from animal by-products, such as those derived from the human food chain, could benefit dog health while contributing to the petfood industry’s economic and environmentally sustainable growth^1^. Protein hydrolysates comprise low molecular weight peptides and free amino acids with several reported in vitro functional properties, such as antioxidant, anti-microbial, anti-inflammatory, and immunomodulatory^2^being also reported to modulate gut microbiota composition^3^.

Protein hydrolysates are commonly used in petfood, especially to prevent allergic reactions in sensitive dogs^4^. However, studies evaluating diets with protein hydrolysates have shown inconsistent effects on gut microbiota, immune response and hematological and biochemical parameters in dogs. For instance, Beagle dogs fed diets containing up to 15% of a commercial mix of black soldier fly larvae hydrolysate and microalgae-like *Schizochytrium* sp. during 28 days had decreased plasma concentrations of the pro-inflammatory cytokine interleukin (IL)-8, triglycerides and total cholesterol, and increased immunoglobulins (Ig) A and G, and albumin levels^5^. Greater levels of fecal IgA were observed with Beagle dogs fed diets including chicken hydrolysate at 25% for 28 days, but not with 25% of chicken liver and heart hydrolysates^6^. German Shepherd dogs supplemented with 0.3% of hydrolyzed yeast *Saccharomyces cerevisiae* for 42 days exhibited an increase in the abundance of fecal bifidobacteria (at 14th day), lactic acid bacteria (at 42nd day) and clostridia (at 42nd day), and an increase in the serum aspartate aminotransferase at 28 days^7^. Including up to 15% shrimp hydrolysate from *Litopenaeus vannamei* in diets of Beagle dogs over a 10-day feeding trial affected the abundances of *Oscillosperaceae*, Bacillota (formerly Firmicutes), and *Lactobacillus* in the fecal microbiota^8^. Conversely, 20% dietary inclusion of pink salmon hydrolysate have failed to demonstrate significant alterations in the immune response of Pointer dogs in a 26-day feeding trial^9^. No variations in fecal microbiota, immune response and hematology were observed in Beagle dogs fed with 25.8% (as fed basis) of hydrolyzed chicken liver for 45 days^10^. The source of protein hydrolysate, the duration of the study, and the specific breed of dogs used might contribute to these conflicting results.

Despite the effects of shrimp hydrolysate that have been investigated in mice and aquaculture, to the best of the authors’ knowledge, there is no information available on the immune response in dogs, and limited data on its effects on microbiota. In mice, a shrimp hydrolysate from *Penaeus chinensis* has been shown to enhance macrophage activation, phagocytosis, the levels of the cytokines interferon-gamma (IFN-γ) and IL-2, and the levels of the antibodies IgA and IgM^11^ and to decrease gut pathogenic bacteria abundance^12^. In mice exposed to chronic stress, shrimp hydrolysate derived from the heads of unidentified species has been shown to restore fecal short-chain fatty acid levels and improve gut microbiota by modulating alpha diversity and maintaining microbiota distribution^13^. Shrimp hydrolysate from *L. vannamei* has been shown to benefit different fish species. In red seabream, it has been shown to increase hemoglobin and hematocrit levels^14^decrease glucose levels, and improve innate immunity by enhancing the lysozyme activity and survival rates of fish infected with *Edwardsiella tarda*^15^. In seabass, it positively influenced the survival of fish affected by an epizootic outbreak, with additional benefits in the non-specific immune responses, such as in the lysozyme, alternative complement and bacteriolytic activities^16^. Moreover, the hydrolysis of *Penaeus monodon* with alkaline protease has reduced in vitro IgE reactivity to tropomyosin^17^.

Building on previous research that evaluated the effects of dietary inclusion of 5% shrimp hydrolysate on diet palatability and digestibility, fecal characteristics, coat quality and oral volatile sulfur compounds of healthy adult Beagle dogs^18^this study focused on assessing its impact on the hematological parameters, serum chemistry profile, innate and adaptive immune function, and fecal microbiota composition.

## Results

### Hematological and biochemical blood profile

The inclusion of shrimp hydrolysate led to a decrease in the percentage of eosinophils (4.50% vs. 5.51%, *P* = 0.017) and in the levels of glucose (86.4 mg/dL vs. 92.8 mg/dL, *P* = 0.023), and increased the concentration of white blood cells (7.67 × 10^3^/µL vs. 6.71 × 10^3^/µL, *P* = 0.002), platelets (300 × 10^3^/µL vs. 274 × 10^3^/µL, *P* = 0.038), and the percentage of neutrophils (53.2% vs. 56.8%, *P* = 0.036; Table 1 and Table S1). Regarding the time effect, greater percentage of eosinophils in the blood (*P* = 0.003), and greater concentrations of total protein (*P* = 0.001), glucose (*P* < 0.001), and hemoglobin (*P* = 0.013) were observed in week eight. Platelet concentration (*P* = 0.040) and mean platelet volume (*P* = 0.004) were greater in week four, whereas albumin concentration was lower (*P* < 0.001). Concentration of IgE in plasma was lower in week 12 (*P* < 0.001; Table S1). The interaction between week and diet affected the concentrations of the red blood cells (*P* = 0.044) and hemoglobulin levels (*P* = 0.037), with dogs fed the control diet showing higher levels than those fed the experimental diet at week 12, no differences being observed among diets for weeks four and eight (Table 1).

**Table 1:** Hematology, serum chemistry, C-reactive protein, plasma Immunoglobulin E in weeks 4, 8 and 12 in dogs fed control and experimental diets.

Item	Week 4			Week 8			Week 12		SEM^2^	*P* - value		
	Control^1^	Experimental^1^		Control^1^	Experimental^1^		Control^1^	Experimental^1^		Diet	Week	Diet*Week
White blood cells, × 10^3^/µL	6.86	7.09		6.83	7.62		6.45	8.29	0.433	0.002	0.601	0.139
Neutrophils, %	54.7	54.7		50.8	56.2		54.2	59.3	1.93	0.036	0.129	0.168
Lymphocytes, %	35.0	35.8		37.2	32.7		34.9	30.7	2.05	0.183	0.265	0.219
Monocytes, %	5.54	4.91		5.42	5.21		5.79	5.81	0.467	0.437	0.347	0.734
Eosinophils, %	4.80	4.25		6.63	5.50		5.11	3.75	0.500	0.017	0.003	0.671
Red blood cells, × 10^6^/µL	7.10^a^	7.30^a, b,c^		7.43^b, c,d^	7.38^a, b,c^		7.49^c, d^	7.18^a, b^	0.153	0.636	0.068	0.044
Hemoglobin, g/dL	16.6^a^	17.1^a, b,c^		17.6^b, c^	17.4^b, c^		17.7^c^	16.9^a, b^	0.37	0.596	0.013	0.037
Platelets, × 10^3^/µL	301	318		252	294		270	289	17.0	0.038	0.040	0.632
Mean platelet volume, fL	10.1	10.1		9.87	9.88		9.64	9.76	0.192	0.914	0.004	0.650
Total protein, g/dL	5.51	5.61		5.85	6.07		5.78	5.89	0.098	0.106	0.001	0.740
Albumin, g/dL	3.59	3.64		3.81	3.89		3.83	3.82	0.061	0.565	< 0.001	0.657
Globulin, g/dL	1.93	1.97		2.04	2.18		1.95	2.06	0.062	0.067	0.054	0.720
Glucose, mg/dL	87.2	82.6		101	95.3		90.3	81.4	2.60	0.023	< 0.001	0.608
Creatinine, mg/dL	0.764	0.836		0.880	0.793		0.762	0.808	0.0635	0.894	0.456	0.143
Urea, mg/dL	26.5	28.7		38.0	28.0		34.5	28.9	3.55	0.098	0.229	0.172
Alanine aminotransferase, U/L	27.7	36.6		27.2	29.4		30.4	29.3	3.34	0.218	0.471	0.273
Alkaline phosphatase, U/L	43.5	45.7		43.3	45.8		42.3	44.0	2.16	0.396	0.618	0.966
C-reactive protein, µg/mL	7.37	8.23		8.41	9.09		5.19	9.01	1.054	1.22	0.234	0.318
Immunoglobulin E, µg/mL	216	195		241	177		55.9	71.3	26.1	0.438	< 0.001	0.135

^1^Control: complete diet without the inclusion of shrimp hydrolysate; Experimental: control diet with 5% of shrimp hydrolysate in replacement of wheat gluten.

^2^SEM: Standard error of the mean.

^a, b,c, d^Means with different superscript letters in the same row are significantly different (*P* < 0.05).

### Serum cytokine, chemokine, and growth factor concentrations

Concentrations of IFN-γ, IL-10, IL-6, nerve growth factor-beta (NGF-β), and tumor necrosis factor-alpha (TNF-α) were below the detection limits (8.42 pg/mL, 6.47 pg/mL, 16 pg/mL, 4.98 pg/mL and 5.22 pg/mL, respectively). Diet and week did not affect the production of Il-12/IL-23p40, IL-8, IL-2, stem cell factor (SCF), monocyte chemoattractant protein-1 (MCP-1), and vascular endothelial growth factor A (VEGF-A; Table 2 and Table S2). The interaction between diet and week affected the production of IL-8 (*P* = 0.038) that was significantly lower at week eight (539 pg/mL) and higher at week 12 (964 pg/mL) in dogs fed the control diet (Table 2). The interaction between diet and week also affected the production of SCF (*P* = 0.027), with lower values being observed at weeks four (29.6 pg/mL) and eight (33.8 pg/mL) and a higher value at week 12 (50.6 pg/mL) in dogs fed the control diet (Table 2). No differences were observed for IL-8 and SCF among weeks in dogs fed the experimental diet.

**Table 2:** Concentration of cytokines, chemokine, and growth factor in serum in weeks 4, 8 and 12 of dogs fed the control and experimental diets.

Item	Week 4			Week 8			Week 12		SEM^2^	*P* - value		
	Control^1^	Experimental^1^		Control^1^	Experimental^1^		Control^1^	Experimental^1^		Diet	Week	Diet*Week
Il-12/IL-23p40, pg/mL	666	1209		749	1223		963	1182	328.8	0.371	0.240	0.115
IL-8, pg/mL	774^a, b^	783^a, b^		539^a^	877^a, b^		964^b^	709^a, b^	144.3	0.862	0.502	0.038
IL-2, pg/mL	28.2	95.9		39.3	104		62.0	97.4	49.90	0.161	0.901	0.899
SCF, pg/mL	29.6^a^	43.9^a, b^		33.8^a^	47.1^a, b^		50.6^b^	41.7^a, b^	15.64	0.750	0.214	0.027
MCP-1, pg/mL	106	103		113	99.0		90.3	98.4	10.17	0.713	0.366	0.464
VEGF-A, pg/mL	6.00	5.62		5.47	5.08		5.85	8.36	1.016	0.622	0.059	0.103

^1^Control: complete diet without the inclusion of shrimp hydrolysate; Experimental: control diet with 5% of shrimp hydrolysate in replacement of wheat gluten.

^2^SEM: Standard error of the mean.

^a, b,c, d^Means with different superscript letters in the same row are significantly different (*P* < 0.05).

Abbreviations: IL, interleukin; SCF, stem cell factor; MCP-1, monocyte chemoattractant protein-1; VEGF-A, vascular endothelial growth factor A.

### Reactive oxygen species production

**Fig. 1:**
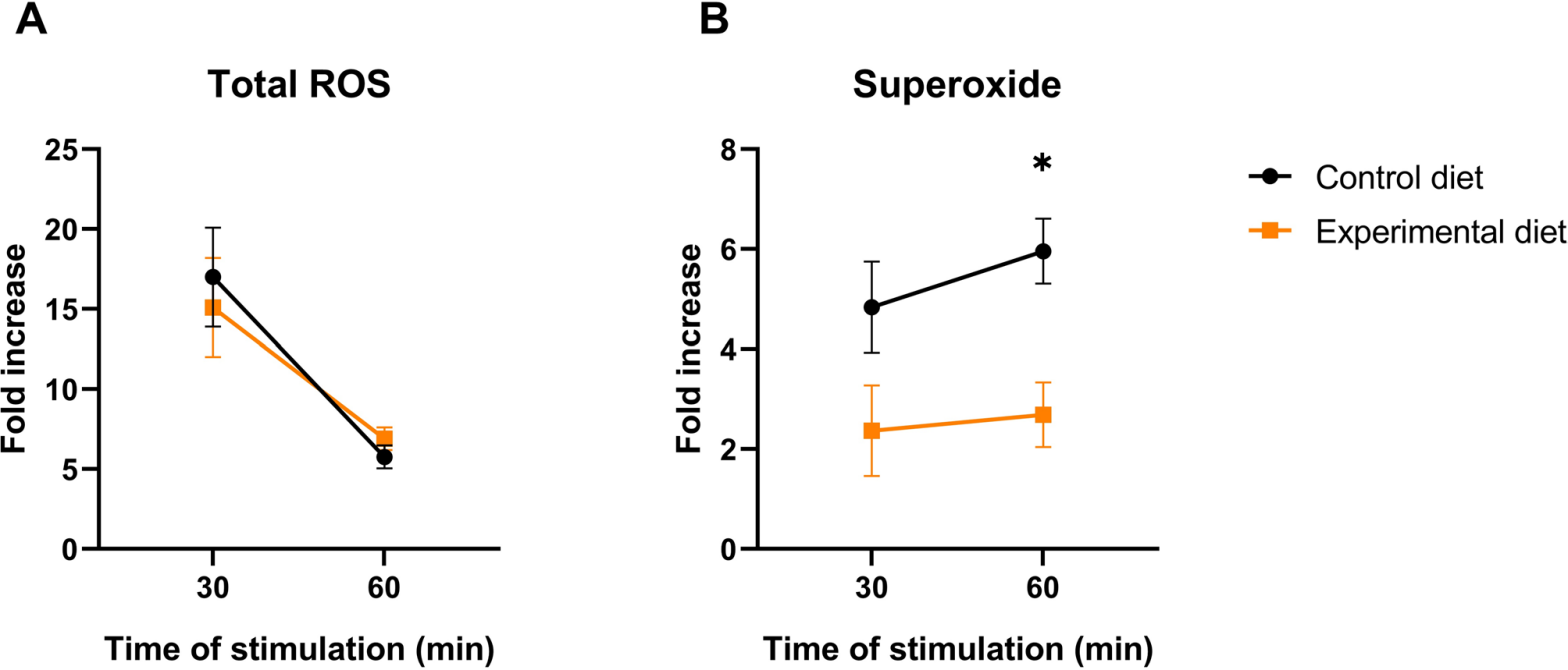
Reactive oxygen species (ROS) production evaluated by flow cytometry. (**A**) Fold increase in the production of total ROS; (**B**) Fold increase in the production of superoxide in cells stimulated with phorbol myristate acetate for 30 and 60 min over the basal production (non-stimulated) in control diet and experimental diet. Bars correspond to mean plus standard error of the mean. * *P* < 0.05.

Cells of both control and experimental diets groups produced greater amounts of total reactive oxygen species (ROS) after 30 min of phorbol myristate acetate (PMA) stimulation than after 60 min (Fig. 1A and Table S3). Conversely, superoxide production was lower after 30 min than after 60 min, with the control diet showing greater levels of production of superoxide after 60 min of PMA stimulation compared to the experimental diet (*P* = 0.002; Fig. 1B). The interaction between diet and week affected the production of total ROS (*P* = 0.030) and of superoxide (*P* = 0.030) after 60 min of PMA stimulation, with values being significantly higher at week 12 regardless of the diet (Table S3).

### Lymphocyte proliferation and cytokine production

**Fig. 2:**
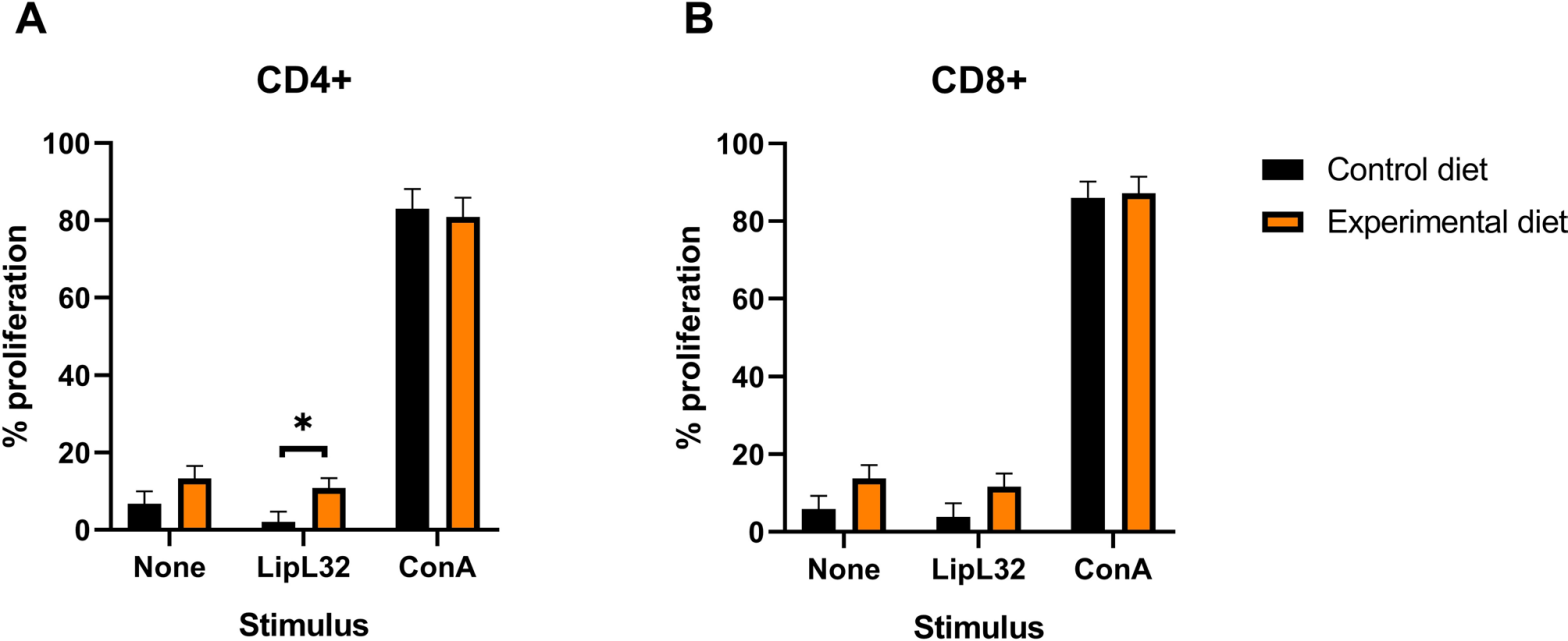
T lymphocyte proliferation evaluated by flow cytometry. (**A**) Percentage of CD3^+^CD4^+^ cells; (**B**) Percentage of CD3^+^CD8^+^ cells, from dogs fed control diet and experimental diet, that proliferated at least once when non-stimulated (None) or in response to recombinant antigen from *Leptospira interrogans* (LipL32) and concanavalin A (ConA). Bars correspond to mean plus standard error of the mean. * *P* < 0.05.

The percentages of proliferation of CD3^+^CD4^+^ and CD3^+^CD8^+^ cells stimulated with concanavalin A (ConA) were not affected by the diet. The experimental diet induced more extensive proliferation of CD3^+^CD4^+^ cells in response to recombinant antigen LipL32, when compared to the control diet (10.8% vs. 2.07%, respectively, *P* = 0.020), and no diet effects were observed in the proliferation of CD3^+^CD8^+^ cells stimulated with this antigen (Fig. 2).

The levels of IL-17, IFN-γ, TNF-α and IL-10 in culture supernatants of non-stimulated peripheral blood mononuclear cells (PBMC) or stimulated with LipL32 were below the detection limits (62.5 pg/mL, 31.3 pg/mL, 31.3 pg/mL and 15.6 pg/mL, respectively). Regarding the time effect, the PBMC stimulated with ConA produced increased levels (*P* < 0.05) of IL-17, TNF-α, and IL-10, and decreased levels of IFN-γ (*P* = 0.011; Table S4) in week 12. No effect of diet and of interaction between diet and week were found in the production of IL-17, IFN-γ, TNF-α and IL-10 in cells stimulated with ConA (Table 3).

**Table 3:** Concentration of cytokines after lymphocyte stimulation with Concanavalin A in weeks 4, 8 and 12 of dogs fed the control and experimental diets.

Item	Week 4			Week 8			Week 12		SEM^2^	*P* - value		
	Control^1^	Experimental^1^		Control^1^	Experimental^1^		Control^1^	Experimental^1^		Diet	Week	Diet*Week
IL-17, pg/mL	470	334		1284	1202		2177	1422	509.3	0.405	0.027	0.752
IFN-γ, pg/mL	2762	1626		1586	1883		1308	875	471.1	0.452	0.011	0.122
TNF-α, pg/mL	25.2	29.4		18.6	19.7		47.8	35.3	5.90	0.594	0.004	0.317
IL-10, pg/mL	135	72.7		156	148		289	211	44.27	0.124	0.002	0.729

^1^Control: complete diet without the inclusion of shrimp hydrolysate; Experimental: control diet with 5% of shrimp hydrolysate in replacement of wheat gluten.

^2^SEM: Standard error of the mean.

Abbreviations: IFN-γ: interferon-gamma; IL, interleukin; TNF-α: tumor necrosis factor alpha.

### Production of IFN-γ and TNF-α by CD4^+^ and CD8^+^ T cells and Foxp3 in CD4^+^

**Fig. 3:**
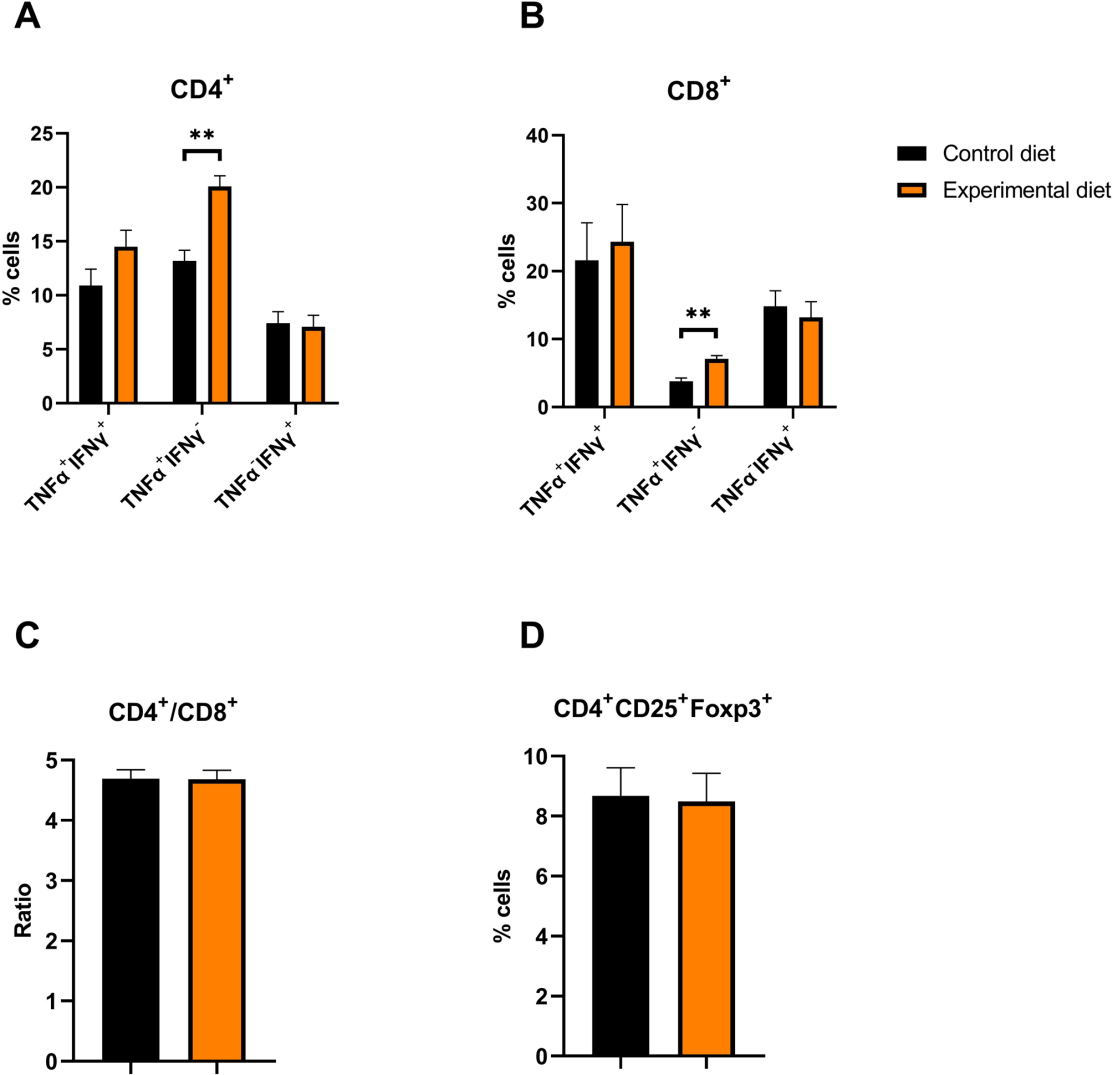
Intracellular cytokine measurement by flow cytometry. (**A**) Percentage of CD3^+^CD4^+^ cells expressing interferon-gamma (IFN-γ), tumor necrosis factor-alpha (TNF-α) and both cytokines; (**B**) Percentage of CD3^+^CD8^+^ cells expressing IFN-γ, TNF-α and both cytokines; (**C**) Ratio of CD3^+^CD4^+^ and CD3^+^CD8^+^ cells calculated from the percentage of CD4^+^ and CD8^+^ T cells; (**D**) Percentage of CD3^+^CD4^+^CD25^+^ cells expressing Foxp3 from dogs fed control diet and experimental diet. Bars correspond to mean plus standard error of the mean. ** *P* < 0.01.

The inclusion of shrimp hydrolysate did not influence the percentage of CD4^+^ and CD8^+^ T cells single IFN-γ producers (Fig. 3A), IFN-γ and TNF-α double producers (Fig. 3B), and the CD4^+^/CD8^+^ T cell ratio (Fig. 3C). However, it positively influenced the CD4^+^ TNF-α T cells single producers (from 13.1%, in dogs fed the control diet, to 20.2%, in those fed the experimental diet, *P* < 0.001), and the CD8^+^ TNF-α T cells single producers (3.78% and 7.09% for dogs fed the control and the experimental diet, respectively, *P* < 0.001; Fig. 3A and B). Regardless of the diet, the percentage of CD4^+^ T cells was greatest in week 4 (63.9%, *P* = 0.050; Table S5). The CD4^+^ T cells double producers of IFN-γ and TNF-α and single producers of TNF-α presented the greatest values in week 12 (15.2%, *P* = 0.002, and 20.3%, *P* < 0.001, respectively), while CD4^+^ T cells single producers of IFN-γ presented the greatest value in week eight (8.62%, *P* < 0.001). CD8^+^ T cells double producers of IFN-γ and TNF-α presented the greatest value in week 12 (32.7%, *P* < 0.001). No differences were found in the percentage of CD4^+^CD25^+^Foxp3^+^ T cells between diets (Fig. 3D), over time and in the interaction of diet and week.

### Fecal IgA and microbiota

**Fig. 4:**
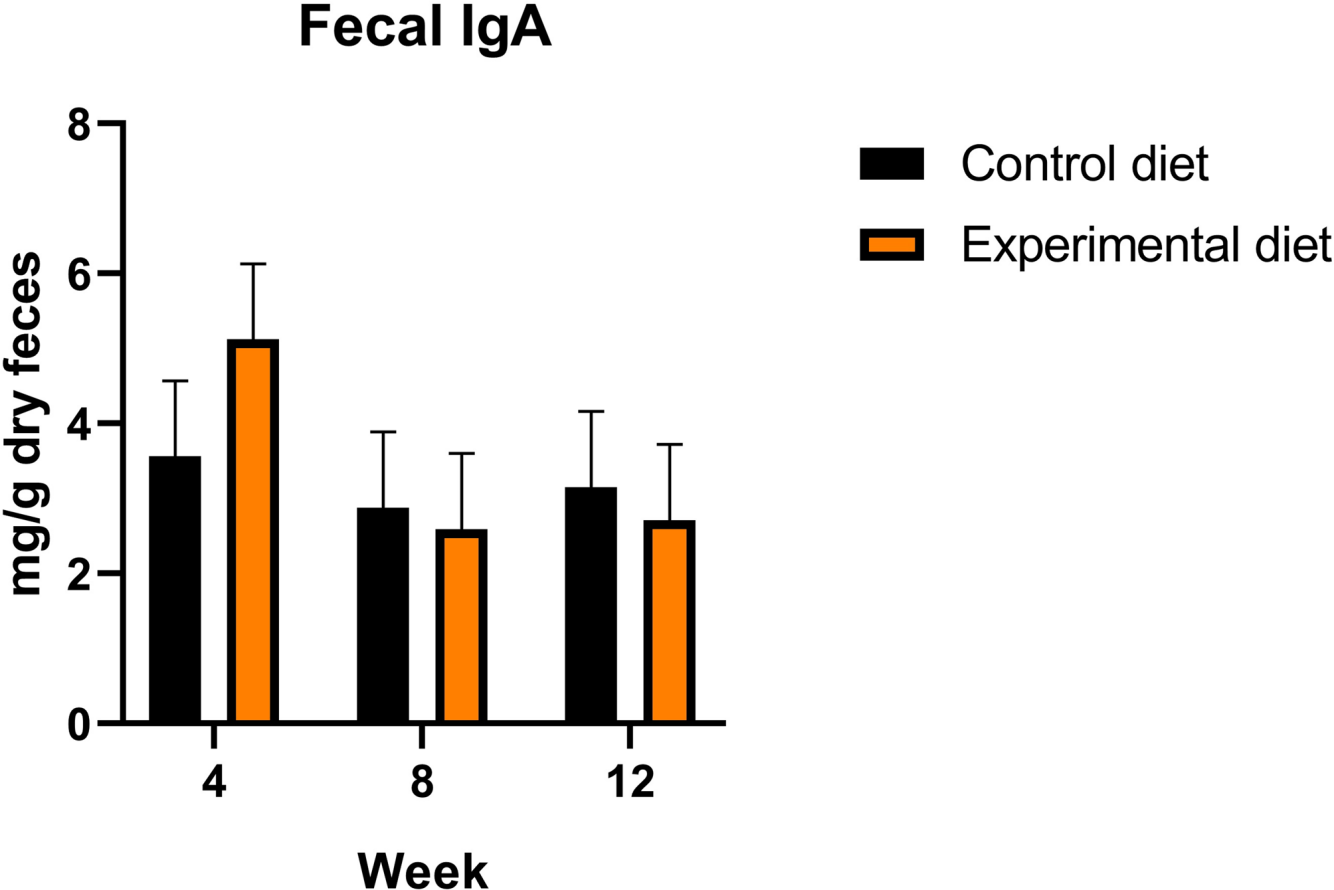
Fecal immunoglobulin A (IgA) concentrations in weeks 4, 8 and 12 in dogs fed control diet and experimental diet. Bars correspond to mean plus standard error of the mean.

**Fig. 5:**
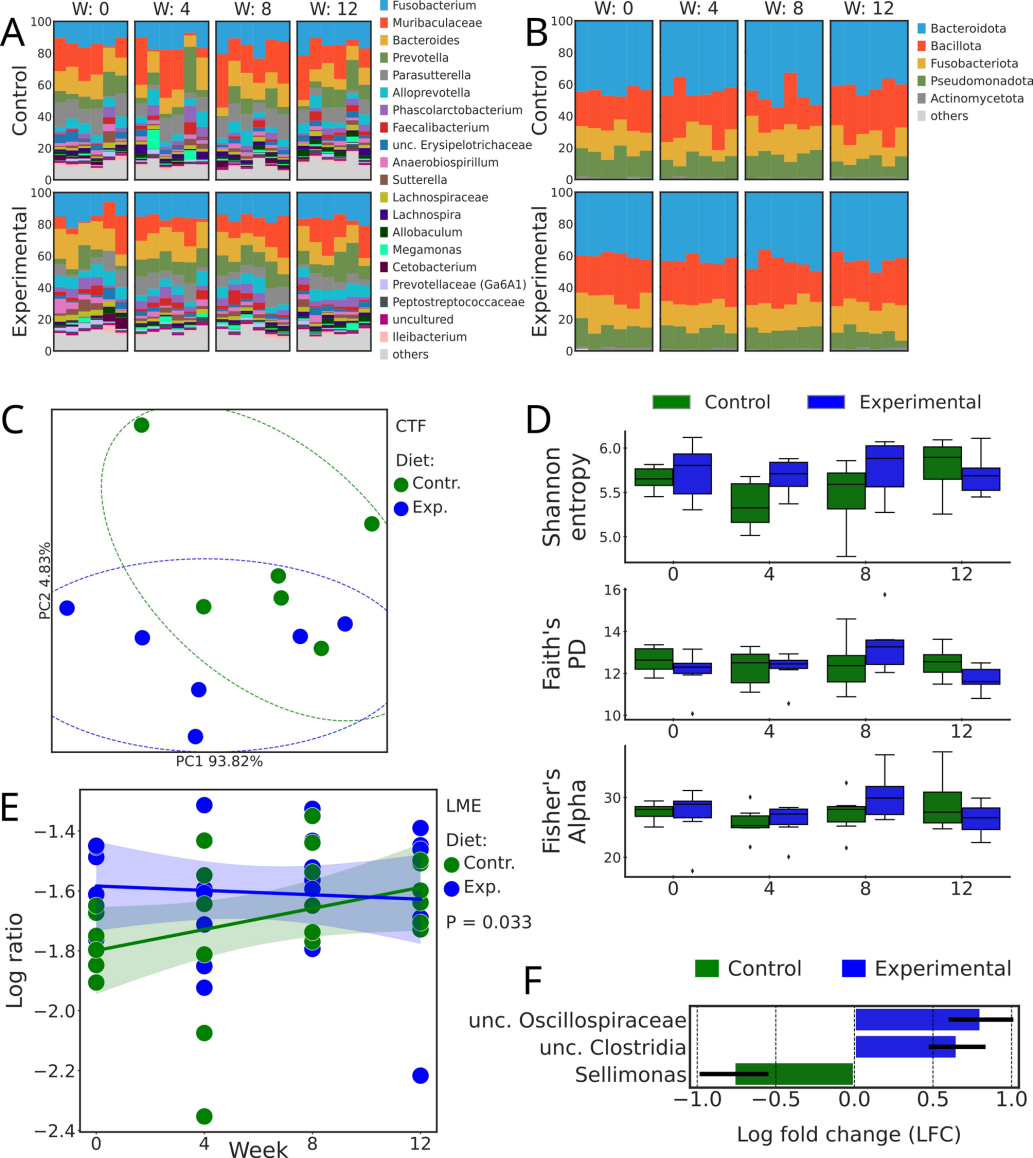
Bacterial relative abundances, composition and diversity. (**A**) Taxonomy barplots at the genus level of dogs fed control and experimental diets in weeks 0, 4, 8, and 12. If genus level was not assigned, the last available taxonomy rank was used for the label; (**B**) Taxonomy barplots at the phylum level of dogs fed control and experimental diets in weeks 0, 4, 8, and 12; (**C**) Beta diversity metrics. Compositional Tensor Factorization (CTF) distance of fecal bacteria of dogs fed control and experimental diets. Each dot represents one dog fed on either diet; (**D**) Alpha diversity metrics. Bloxplots of Shannon entropy Faith’s PD and Fisher’s Alpha indices of fecal bacteria of dogs fed control and experimental diets; (**E**) Linear mixed-effects analysis based on log-ratios of fecal bacteria of dogs fed control and experimental diets; (**F**) Differentially abundant genera (*P*-adj < 0.05), according to ANCOM-BC. The fecal bacteria of dogs fed the experimental diet were compared to those of the control diet.

Fecal IgA concentration was not affected by diet, week, and their interaction (Fig. 4). Regardless of the diet, *Fusobacterium* was the most abundant genus, followed by genus pertaining to *Muribaculaceae* and genus *Bacteroides* (Fig. 5A). Across all the samples, most of the features were assigned to the phylum Bacteroidota (formerly Bacteroidetes), followed by Bacillota (Fig. 5B). Beta diversity metrics indicate a clear separation of bacterial communities between diets, by using Compositional Tensor Factorization (CTF) distance (Fig. 5C). However, when Bray-Curtis distances were compared at each time point, no differences between the diets were detected. At the same time, linear mixed-effects analysis performed on log-ratios of feature loadings of abundances that contributed to diets separation confirmed a separation between control and experimental diets (*P* = 0.033, r^2^ = 0.215; Fig. 5E). Regarding alpha diversity, no differences were observed between diets on Shannon entropy, Faith’s phylogenetic diversity and Fisher’s Alpha through Kruskal-Wallis and Wilcoxon tests between diets within weeks (Fig. 5D). The experimental diet led to an increased abundance of genera pertaining to *Oscillospiraceae* and Clostridia, while the abundance of *Sellimonas* decreased (*P* < 0.05; Fig. 5F).

## Discussion

The incorporation of animal by-products in pet nutrition has been a subject of growing interest due to its potential impact on animal health and industry sustainability^19^. From a nutritional standpoint, hydrolyzed protein from animal by-products, which comprise smaller peptides and amino acids, may offer essential nutrients and functional properties that can benefit dogs’ health^20^. This study aimed to evaluate the effects of the dietary inclusion of 5% shrimp hydrolysate on hematological, serum chemistry, immunological parameters, and fecal microbiota of healthy adult Beagle dogs.

Overall, the blood profiles of dogs showed values within the established reference ranges for healthy adult dogs^21,22^. The dietary inclusion of shrimp hydrolysate led to an increase in neutrophils, platelets and white blood cells, suggesting that shrimp hydrolysate might play a role in modulating the immune function and supporting a healthy bone marrow function^23^. Furthermore, the inclusion of shrimp hydrolysate led to a decrease in eosinophils concentration, but no variations in IgE were observed among diets. Eosinophils are known to play a role in allergic reactions and combating certain infections. Their differentiation and activation can be mediated by cytokines, such as IL-5 and IL-3, chemokines, prostaglandin D2, and indirectly be influenced by the IgE pathway^24^. The levels of eosinophils observed in the present study were within the normal range values for dogs (0–9%^21^), likewise the levels of IgE in blood (25–410 µg/mL^25^). Nevertheless, a reduction in the concentration of eosinophils suggests that shrimp hydrolysate may modulate the immune system, potentially decreasing inflammation or allergic responses. This is consistent with the finding that shrimp hydrolysate did not affect IgE-mediated allergic responses in the dogs. Previous research has shown that the dietary inclusion of chicken liver hydrolysate induced a decrease in the levels of eosinophils and IgE in the plasma of dogs over a 45-day feeding trial^10^. The IgE levels decreased in week 12 in dogs fed either diet, whereas the eosinophils levels were lower in weeks four and 12.

The inclusion of shrimp hydrolysate led to a significant decrease in glucose levels in the blood compared to the control diet, despite all values remaining within the normal range for healthy dogs (76–119 mg/dL^21^). Fish hydrolysates have been demonstrated to lower glucose levels and regulate hyperglycemia in vitro, in murine models, and in human subjects^26^. Furthermore, reduced glucose levels were observed in red seabream fed diets containing shrimp hydrolysate from *L. vannamei*^15^. Little is known about the effect of hydrolyzed proteins in blood glucose levels in dogs. In a study using an in vitro gastrointestinal digestion model for dogs, it was observed that, compared to raw tilapia, its hydrolysate enhanced the secretion of active glucagon-like peptide-1 (GLP-1), a hormone involved in blood glucose regulation, and improved the inhibition of dipeptidyl peptidase-IV enzymatic activity in Caco-2 cells, crucial in degrading GLP-1^27^. Moreover, diets containing hydrolyzed yeast *S. cerevisiae* have been shown to increase blood glutathione concentration, an antioxidant which may protect cells from oxidative damage caused by ROS^28^. The excessive production of superoxide radicals is considered the main mechanism responsible for tissue damage in diabetes mellitus^29^and superoxide production may stimulate insulin release through the metabolism of branched-chain keto-acids in mitochondria^30^. Additionally, high blood glucose levels increase the generation of ROS in both mitochondria and cytosol, which contributes to the development of various diabetes-related pathologies^31^. Therefore, the decreased levels of blood glucose herein observed might be associated with the decreased production of superoxide compared to dogs fed the control diet, suggesting that lowering superoxide production could have led to upregulation of glucose metabolism. These results agree with the reported in vitro antioxidant activity of shrimp hydrolysate^8^which possibly helped reduce superoxide production by neutralizing free radicals. However, no differences were found in total ROS production between the control and experimental groups.

The interaction between diet and week affected serum concentrations of IL-8 and SCF, with the lowest and greatest values observed in weeks eight and 12 in dogs fed the control diet. In dogs fed the experimental diet, the concentrations remained unaltered over time. IL-8 is a chemokine produced by various immune cells and it plays important roles in a wide variety of functions, such as recruiting neutrophils, basophils and T cells during immune responses to infection, inflammation, and white blood cells activation^32^. Stem cell factor is a cytokine that binds to the c-kit, a tyrosine kinase receptor, and interacts with other cytokines, protecting the viability of hematopoietic cells, while also inducing their proliferation and differentiation^33^. The increase of both IL-8 and SCF could be linked, as previously demonstrated^34^potentially resulting from the production of IL-8 by hematopoietic progenitors cells^35^.

No differences were found between control and experimental groups in the production of cytokines by lymphocytes stimulated with ConA. Similarly, gene expression of IL-10 and IFN-γ in dogs were not affected by the dietary inclusion of pink salmon hydrolysate^9^. Additionally, dog diets including black soldier fly larvae hydrolysate and *Schizochytrium* sp. did not affect the production of TNF-α in non-stimulated cells, while the levels of IL-8 in plasma decreased^5^. Although the levels of IL-8 in serum were similar between diets, the inclusion of shrimp hydrolysate tended to result in decreased IL-8 levels, suggesting a possible anti-inflammatory effect. Lymphocytes stimulated with ConA produced increased levels of IL-17, TNF-α and IL-10 over time, whereas the production of IFN-γ decreased. The levels of IL-17 and IFN-γ can exhibit inverse or corresponding patterns in immune responses due to complex regulatory networks^36,37^and diet might influence their regulation with important effects in autoimmune diseases^38^. Moreover, TNF-α can promote a reduction of Th1 cells producing IFN-γ and an increase of Th17 cells secreting IL-17^39^, and an increment of the anti-inflammatory cytokine IL-10^40^. IL-10 can inhibit pro-inflammatory responses of innate and adaptive immune cells by suppressing the production of various cytokines, such as IFN-γ and TNF-α^41^. It has also been shown to play an important role in the homeostasis of intestinal mucosal^42^.

Diet did not influence lymphocyte proliferation, except for the greater proliferation of CD4^+^ T cells stimulated with LipL32 from dogs fed shrimp hydrolysate compared to the control diet. LipL32 is a protein present in outer membrane of leptospires that may induce a proliferative response by memory CD4^+^ T cells^43^. Dogs that participated in the study were annually vaccinated for leptospires. Recently, it has been shown that the percentage of central memory CD4^+^ T cells and proliferation of CD4^+^ T cells in response to different *Leptospira* serovars increased in dogs after vaccination^44^. Diet plays an important role in the regulation of memory T cells, opening the possibility of developing and implementing diet-based therapies that can be used to attain more effective vaccination strategies^45^. Therefore, the results might indicate a positive effect of shrimp hydrolysate in the generation of memory CD4^+^ T cells, which could lead to a more effective immune response against leptospiral infections in dogs. Furthermore, lymphocyte proliferation has been observed in PBMC isolated from blood of dogs with suspected food hypersensitivity when cultured with extracts of commercially hydrolyzed canine diets^46^. Despite no research on lymphocyte proliferation of healthy dogs fed protein hydrolysates has been conducted, hydrolysates from Alaska pollock frame, oyster and *Paphia undulata* were shown to enhance lymphocyte proliferation of cells isolated from mouse spleens^47–50^.

No differences between control and experimental groups were found in the percentage of CD4^+^ and CD8^+^ T cells, and in their ratio. Similar results were observed in dogs fed diets with hydrolyzed yeast inclusion^7^. Dietary inclusion of shrimp hydrolysate promoted the increment in CD4^+^ and CD8^+^ T cells single producers of TNF-α. While TNF-α plays an important role in the defense against pathogens^51^high circulating levels in blood have been associated with the development of insulin resistance, diabetes and cardiovascular disease^52^. However, experiments conducted in ob/ob mice and TNFR1/R2 double knockout mice have demonstrated that increasing TNF-α levels can enhance glucose homeostasis^53^. The authors suggest that TNF-α plays a more complex role in glucose regulation than previously assumed, through an alternative receptor-independent mechanism that positively influences glucose homeostasis. Therefore, further research is required to understand whether the increase of CD4^+^ and CD8^+^ T cells single producers of TNF-α in dogs fed shrimp hydrolysate may be beneficial to dogs’ health. The levels of CD4^+^CD25^+^Foxp3^+^ regulatory T cells remained similar among diets, which indicates no alterations in the general immune homeostasis of both groups of dogs, probably influenced by a healthy gut environment with either diet^54^.

Immunoglobulin A is a secretory immunoglobulin present, among others, in the intestinal mucosa, protecting it from pathogens that can be used as an inflammatory biomarker^55^. The levels of IgA might be influenced by diet^56,57^. Supplementing the diet with shrimp hydrolysate did not affect fecal IgA levels. There is a lack of research studying the effects of dietary inclusion of protein hydrolysates in IgA levels in dog feces. Studies on the effects of marine protein hydrolysates on intestinal IgA levels in other animal species have generated conflicting results. The IgA levels in the middle and distal intestine of turbot were not influenced by the inclusion of fish protein hydrolysate in diets^58^whereas feeding mice with fish protein hydrolysate led to an increase in IgA concentrations in the small intestine^59^. Additionally, IgA helps to regulate the intestinal microbiota, such as their colonization, invasion, growth, and motility^60^. In turn, the microbiota stimulates the production of IgA, and, unlike classical immunological memory responses, the immune system produces IgA antibodies that are specific for gut microbial species^61^. Furthermore, similar to human patients, also higher concentrations of IgA^+^ bacteria, which specifically bound to IgA, were observed in dogs with inflammatory bowel disease compared to healthy dogs^62^.

The greatest relative abundance of genus found in both diets pertained to phyla Bacteroidota and Bacillota. These phyla abundance is in accordance with data reported in dog feces^63^. The four most predominant genera found (*Fusobacterium*, *Muribaculaceae*, *Bacteroides*, *Prevotella*) were previously observed in Beagle dogs fed diets with comparable macronutrients composition^64^suggesting that microbiota composition is mainly a consequence of the amount of macronutrients that comprise diets than of the ingredients^10^. *Fusobacterium* was the most predominant genera found in both diets, agreeing with a previous study in which dogs fed diets without and with chicken liver hydrolysate^10^. According to our previous findings^8^the inclusion of shrimp hydrolysate led to an increment in the abundance of genus belonging to *Oscillospiraceae*. *Oscillospira*, a genus from *Oscillospiraceae*, has been suggested to be a great probiotic candidate in future treatments, such as in obesity and chronic inflammation^65^. Increased production of short-chain fatty acids beneficial to animal health^66–68^such as acetate, butyrate, propionate and valerate have been associated with the presence of *Oscillospira* in the gut^65,69^. However, a decrease in fecal butyrate levels was observed in dogs fed shrimp hydrolysate, compared to a diet without hydrolysate inclusion^18^. Clostridia, a class of Bacillota, was also increased in feces of dogs fed diets supplemented with shrimp hydrolysate. Different species of this class have been associated with various disorders of the gastrointestinal tract of dogs^70^. Previous research has suggested an association between improved digestibility of protein and increased abundance of *Clostridiaceae* in dogs^71^being bacteria of the *Clostridium* genus crucial in the fermentation of amino acids, such as lysine and proline^72^. Although digestibility of amino acids was not analyzed in the current trial, no alterations in the digestibility of protein were observed among control and experimental diets^18^. Nevertheless, because the hydrolysis process breaks down proteins into small peptides, bacteria from Clostridia might be able to metabolize these peptides in the intestinal tract, prompting their growth. *Sellimonas* decreased in dogs fed the experimental diet. Higher abundances of *Sellimonas* and lower abundances of *Oscillospiraceae* have been observed in human patients with inflammatory bowel disease, compared to healthy humans^73^. However, in dogs diagnosed with inflammatory bowel disease, a decrease in the relative abundance of *Sellimonas* and *Oscillospiraceae* UCG–005 has been observed^74^. Further studies are needed to understand the role of *Sellimonas* in dog gut.

Overall, the current study has shown the potential of shrimp hydrolysate to be included in dog diets, by maintaining the general health of dogs, namely their immune function and fecal microbiota, while promoting the sustainability of the petfood industry. The findings suggest that it has an immunomodulatory role, evidenced by increased neutrophils, white blood cells, and platelets, alongside decreased eosinophil levels, indicating its potential for inclusion in hypoallergenic diets. The observed reduction in blood glucose levels and superoxide production in PBMCs suggests its potential as a supplement in diets for diabetic dogs. Furthermore, the increased proliferation of CD4^+^ T cells in response to LipL32 indicates a potential benefit in vaccination. Additionally, shrimp hydrolysate positively impacted the relative abundance of genera pertaining to *Oscillospiraceae* and Clostridia, suggesting it could function as a prebiotic and enhance amino acid digestibility, suggesting its potential in gastrointestinal diets. Future studies should explore the underlying mechanisms responsible for these outcomes. Understanding these mechanisms will provide further insights into the role of shrimp hydrolysate in promoting health and its potential applications in hypoallergenic and gastrointestinal diets, diets for diabetic dogs, and immunomodulatory therapies.

## Methods

The trial was approved by the Animal Ethics Committee of the School of Medicine and Biomedical Sciences, University of Porto, licensed by the Portuguese General Directorate of Food and Veterinary Medicine (Permit N° 0421/000/000/2021). Animal handling and procedures were performed by trained scientists in laboratory animal science (FELASA, Category C) in accordance with the European Union Directive 2010/63/EU on the protection of animals used for scientific purposes. This study was carried out in agreement with the ARRIVE guidelines.

### Animals, diet and experimental design

Details on animals, diets and experimental design of the trial were earlier reported^18^. Briefly, 12 adult Beagle dogs, six females and six males, 4.5 ± 0.65 years-old, 12.4 ± 2.53 kg of body weight (BW), and body condition score with a median (interquartile range) of 5.0 (1) out of 9^75^, were selected for this study. Dogs were kept in pairs within environmentally enriched boxes in the university kennel. Each dog received its daily food ration individually, divided into two meals at 8:30 a.m. and 5:00 p.m. Daily food allowance was calculated according to requirements of metabolizable energy (ME) and ideal BW^76^following the equation ME (kcal/d) = 110 × BW^0.75^. Fresh water was provided *ad libitum*.

Two extruded isoproteic diets were formulated to meet the nutritional requirements of adult medium dogs^76^using the same ingredients, except for the inclusion of 5% (w/w) shrimp hydrolysate (experimental diet) in replacement of wheat gluten (control diet; Table S6), a high-level protein source^18^ commonly used in petfood. The level of inclusion was chosen based on results previously published^8^ and due to practical and economic considerations for the petfood industry. The shrimp hydrolysate (Symrise Aqua Feed, Elven, France) was obtained by enzymatic hydrolysis of heads and cephalothoraxes of *L. vannamei*^77^. A detailed characterization of the diets was earlier reported^18^. The feeding trial was performed according to a complete randomized block design with 12 dogs distributed into six blocks of two dogs each, according to sex and BW. Within each block, one dog was randomly assigned to one of the two diets, totaling six dogs per diet. The study lasted 12 weeks, comprising four time-points of blood and feces collection at weeks 0, 4, 8 and 12.

### Blood collection and analyses

Blood samples were collected before the morning meal via jugular vein puncture into VACUETTE ETDA (Greiner Bio-one, Kremsmunster, Austria), VACUETTE Lithium Heparin (Greiner Bio-one) and VACUETTE Serum Blood Collection (Greiner Bio-one) tubes. The blood samples were centrifuged at 500 *×* g at 22 °C, for 10 min, to allow the isolation of white blood cells. The plasma was recovered and stored at -80 °C for later IgE quantification. For PBMC isolation, the buffy coat was diluted 1:2 in phosphate buffer saline 1*×* (PBS, Sigma Aldrich, St. Louis, MO, USA). The PBMC were separated by gradient density centrifugation using Histopaque 1.077 (Sigma Aldrich) and washed using PBS. The PBMC were stained with a Tuerk’s solution (Sigma Aldrich) and counted using a Neubauer counting chamber^78^. The PBMC were re-suspended in PBS for ROS assay, and in fetal bovine serum (FBS, heat inactivated South America origin, S181H, BioWest, Nuaillé, France) with dimethyl sulfoxide (25-950-CQC, Corning, Glendale, AZ, USA) at 10% (v/v) to be stored at -80 °C until usage in lymphocyte proliferation and intracellular staining assays^79^. Serum samples were centrifuged at 3000 rpm at 20 °C, for 10 min (Thermo Scientific Heraeus Megafuge 16R, Thermo Fisher Scientific, Carlsbad, MA, USA), for evaluation of serum biochemistry and C-reactive protein, and stored at -80 °C for later cytokine quantification assays.

### Hemogram, serum chemistry, C-reactive protein and plasma IgE

The hemogram was performed using a hematology analyzer (Sysmex XN-V, Norderstedt, Germany) and the serum chemistry using a Roche Cobas c501 analyzer (Roche Diagnostics, Basel, Switzerland). C-reactive protein was assessed in serum by immunoturbidimetry using a Roche Cobas c501 analyzer (Roche Diagnostics) with the Gentian Canine CRP Immunoassay Kit (Gentian Diagnostics, Stockholm, Sweden). IgE levels in plasma were determined using a commercial canine IgE ELISA kit (MyBioSource, San Diego, CA, USA), following manufacturer’s instructions.

### Cytokine, chemokine, and growth factor quantification in serum

Serum samples were thawed and the concentrations of IFN-γ, IL-10, IL-12/IL-23p40, IL-2, IL-6, IL-8 (CXCL8), MCP-1/CCL2, NGF-β, SCF, TNF-α and VEGF-A were determined using the ProcartaPlex Canine Cytokine/Chemokine/Growth Factor Panel 1, 11plex (Invitrogen, Carlsbad, CA, USA), following manufacturer’s instructions. The analysis was performed in the i3S Scientific Platform Bioimaging with the Bio-Plex 200 system with high-throughput fluidics (Bio-Rad, Hercules, CA, USA). All washing steps were carried out with the washing buffer in an automated Bio-Plex Pro Wash Station (Bio-Rad). Data acquisition and analysis were performed on the Bio-Plex 200 system, using the Bio-Plex Manager Software version 6.2 (Bio-Rad).

### Reactive oxygen species production

For the evaluation of ROS production, PBMC (1 × 10^6^ cells/well) were stimulated with 100 nM PMA for 30 and 60 min at 37 °C and 5% CO_2_. Non-stimulated cells were used to set basal ROS production. For the quantification of ROS, cells were stained using the ROS-ID Total ROS/Superoxide Detection Kit (Enzo Life Sciences, Lausen, Switzerland), following manufacturer’s instructions. Data was acquired by flow cytometry using a FACSCanto II system (BD Biosciences, Franklin Lakes, NJ, USA) and analyzed using the FlowJo v10 software (BD Biosciences; Figure S1).

### Lymphocyte proliferation and cytokine measurement

The PBMC were thawed using complete RPMI 1640 Medium (Sigma Aldrich) at 37 °C and left resting overnight at 37 °C and 5% CO_2_. Cell populations were stained with trypan blue (Sigma Aldrich) and counted using a Neubauer counting chamber. For the assessment of lymphocyte proliferation, cells were stained with CellTrace Violet Cell Proliferation Kit (Life Technologies Corporation, Eugene, OR, USA). Cells were plated at 2.5 × 10^4^ cells/well in 96-well plates and incubated for 4 days at 37 °C and 5% CO_2_, with 1 µg/mL of ConA from *Canavalia ensiformis* (C0412, Sigma Aldrich) or 10 µg/mL of recombinant antigen from *Leptospira interrogans* (LipL32, Rekom Biotech, Granada, Spain). Non-stimulated cells were used as negative controls of cell proliferation. Plates were centrifuged at 300 *×* g for 5 min, and supernatants were collected and stored at -80 °C for later cytokine measurements. Cells were stained with the antibodies anti-dog CD3 FITC-conjugate (clone CD3-12, MCA1174F, Bio-Rad), anti-dog CD4 PE-Cy7-conjugate (clone YKIX302.9, 25-5040-42, eBioscience, San Diego, CA, USA) and anti-dog CD8 AlexaFluor700-conjugate (clone YCATE55.9, MCA1039A700, Bio-Rad), and incubated protected from light during 20 min at 4 °C. Propidium iodide was added to tubes prior to acquisition to assess cell viability. UltraComp eBeads (Invitrogen) were used for compensation. Samples were analyzed by flow cytometry using a LSR Fortessa analyzer (BD Biosciences, Franklin Lakes, NJ, USA; Figure S2). Culture supernatants were later thawed to determine the concentration of TNF-α, IFN-γ, IL-10, and IL-17 A, using commercial canine ELISA kits (Canine DuoSet ELISA, R&D Systems, Oxford, UK), according to the manufacturer’s instruction. The colorimetric detection was assessed with a Multiskan EX microplate reader (Thermo Fisher Scientific), equipped with Ascent software (Thermo Fisher Scientific).

### Intracellular staining

Peripheral blood mononuclear cells were thawed, washed in complete RPMI at 37 °C, and left resting overnight at 37 °C and 5% CO_2_. Cells were plated at 1 × 10^6^ cells/well and incubated at 37 °C and 5% CO_2_ for 4 h in the presence of 1× eBioscience Cell Stimulation Cocktail (Invitrogen) and 3 µg/mL of eBioscience brefeldin A (Invitrogen). Cell viability was assessed using eBioscience Fixable Viability Dye (FVD) eFluor 506 (Invitrogen). Samples were first stained with FVD at 1:1000 in PBS, protected from light, for 20 min at 4 °C. After washing with PBS, cells were stained with the antibodies anti-dog CD3 FITC-conjugate (Bio-Rad), anti-dog CD4 PE-Cy7-conjugate (eBioscience), anti-dog CD8 AlexaFluor700-conjugate (Bio-Rad) and anti-dog CD5 PE-conjugate (clone YKIX322.3, 12-5050-42, eBioscience), at pre-titrated dilutions in FACS buffer (PBS, 10 mM of NaN_3_, 2% FBS) and incubated, protected from light, for 25 min at 4 °C. Cells were then washed with PBS and fixed with 2% formaldehyde. For Fcγ receptor nonspecific binding, cells were pre-treated with Canine Fc Receptor Binding Inhibitor Polyclonal Antibody (14-9162-42, eBioscience), for 10 min at room temperature, protected from light. After cell fixation with formaldehyde 2%, cells were permeabilized with permeabilization buffer [0.5% saponin (Sigma Aldrich) in FACS buffer] for intracellular staining with the antibodies anti-bovine IFN-γ Alexa Fluor 647-conjugated (clone CC302, MCA1783A647, Bio-Rad) and anti-human TNF-α eF450-conjugated (clone MAb11, 48-7349-42, eBioscience) that cross-react with canine IFN-γ and TNF-α, respectively^80^. The PBMC were incubated, protected from light, for 30 min at room temperature, washed twice in permeabilization buffer, and transferred into cytometry tubes. Data acquisition was performed by flow cytometry with a LSR Fortessa analyzer (BD Biosciences; Figure S3).

For intracellular staining of the transcription factor Foxp3, PBMC (1 × 10^6^ cells/well) were stained with FVD eFluor 780 (Invitrogen) at 1:1000 in PBS and incubated, protected from light, for 20 min, at 4 °C. The PBMC were washed with PBS and stained with anti-dog CD3 FITC-conjugate (Bio-Rad), anti-dog CD4 PE-Cy7-conjugate (eBioscience), and anti-dog CD25 Super Bright 436-conjugate (clone P4A10, 62-0250-42, eBioscience), protected from light, for 25 min at 4 °C. The PBMC were washed with FACS buffer, fixed with Foxp3 Fixation/Permeabilization solution (eBioscience) for 45 min, and permeabilized using Foxp3 Permeabilization Buffer (eBioscience). The PBMC were pre-treated with Canine Fc Receptor Binding Inhibitor Polyclonal Antibody (eBioscience) and incubated with anti-mouse/rat Foxp3 eF506-conjugate (clone FJK-16s, 69-5773-82, eBioscience), protected from light, for 30 min at room temperature. The PBMC were transferred into cytometry tubes for data acquisition by flow cytometry with a LSR Fortessa (BD Biosciences; Figure S4). UltraComp eBeads were used for antibody-fluorescence compensation.

### Fecal collection and analyses

During two consecutive days at weeks 0, 4, 8, and 12, individual fresh feces were collected immediately after defecation and stored at -20 °C per dog and per week until further analysis. Feces were later thawed and homogenized.

### Fecal IgA extraction and determination

Fecal IgA extraction was performed based on Peters et al. (2004). Briefly, 1 g of thawed and homogenized feces was diluted in 10 mL of extraction buffer (PBS with 0.5% Tween 20, Sigma-Aldrich) and centrifuged at 1500 *×* g at 5 °C for 20 min. Eighty µL of a 25 × concentrated solution of complete EDTA-free Protease Inhibitor Cocktail (04693132001, Roche Diagnostics) were added to 2 mL of supernatant, and centrifuged at 15,000 *x* g at 5 °C for 15 min. Supernatant was stored at -20 °C until further analysis. Fecal IgA concentration was assessed using a commercial canine IgA ELISA kit (Dog IgA ELISA Quantitation Set, E44-104, Bethyl Laboratories Inc., Montgomery, TX, USA), following manufacturer’s instructions. After optimal dilution determination, samples were diluted in 1:300 or 1:400 in dilution buffer. Absorbance was read in a Multiskan EX microplate reader (Thermo Fisher Scientific), equipped with Ascent software (Thermo Fisher Scientific). The analyses were performed in duplicate.

### Fecal microbiota analyses

Fecal DNA was extracted with E.Z.N.A. Stool DNA Kit (Omega Bio-tek, Inc., Georgia), following manufacturer’s instructions. Primers targeting the V4 region of the 16 S rRNA gene (forward: GTGYCAGCMGCCGCGGTAA, reverse: GGACTACNVGGGTWTCTAAT) with attached adapters and barcodes were then used for amplification^82^. The produced sequences were purified, quantified, and homogenized. Qualified libraries were sequenced on an Illumina Novaseq 6000 sequencer. Bioinformatic analyses of microbial data were performed using the Qiime2 pipeline^83^. Primers and adapters were removed from the sequences by the cutadapt^84^. After trimming, reads were denoised and merged by the dada2^85^. Resulted amplicon sequence variants were classified by VSEARCH-based consensus^86^ and pre-fitted sklearn-based classifiers^87^ against the Silva database (v138.1, 16 S 99%)^88^. The reference reads were preprocessed by RESCRIPt^89^. The taxonomic classification of phyla has been updated to reflect the most recent nomenclature.

### Calculations and statistical analysis

The CD4^+^/CD8^+^ ratio was calculated from the percentage of CD4^+^ and CD8^+^ T cells. Data on blood parameters and fecal IgA were analyzed according to a mixed model with repeated measurements, including diet, week, and the interaction between diet and week as fixed effects, block as a random effect, and week in the subject dog as a repeated measure, using the SAS software (2022, release 3.81., SAS Institute Inc., Cary, NC, USA). When significant (*P* ≤ 0.05), multiple comparison of means was conducted by Tukey’s post hoc test. For alpha diversity estimation of fecal microbiota, Shannon’s entropy^90, ^Faith’s phylogenetic diversity^91^ and Fisher’s Alpha^92^ indices were calculated, and for beta diversity, CTF^93^ and Bray-Curtis^94^ distances were used. Alpha diversity metrics were compared by the Wilcoxon test for dependent and the Kruskal-Wallis test for independent samples. Shannon differences were calculated as longitudinal differences of Shannon entropy at weeks 4, 8 and 12 to week 0, which served as baseline. For beta diversity, log ratios of features that contributed to the separation of subjects based on the CTF Principal coordinate analysis plot were extracted by Qurro^95^. Bray-Curtis distances were compared by PERMANOVA test^96^. Shannon differences and log ratios were then analyzed with linear mixed-effects models (LME)^97^. Differentially abundant genera (only for counts of genera with relative abundance ≥ 0.1% and prevalence ≥ 10%) were detected by Ancom-BC^98^.

## Electronic supplementary material

Below is the link to the electronic supplementary material.

Supplementary Material 1

## Data Availability

The data generated and analyzed during the current study are available from the corresponding author on reasonable request. Fecal DNA raw sequences obtained in this study are available at the European Nucleotide Archive (ENA) under accession number PRJEB75174.

## References

[1] Hou, Y., Wu, Z., Dai, Z., Wang, G. & Wu, G. Protein hydrolysates in animal nutrition: Industrial production, bioactive peptides, and functional significance. *J. Anim. Sci. Biotechnol.* **8**, 24. 10.1186/s40104-017-0153-9 (2017). 28286649 10.1186/s40104-017-0153-9 PMC5341468

[2] Sánchez, A. & Vázquez, A. Bioactive peptides: A review. *Food Qual. Saf.* **1**, 29–46. 10.1093/fqsafe/fyx006 (2017).

[3] Wu, S. et al. Bioactive peptides and gut microbiota: candidates for a novel strategy for reduction and control of neurodegenerative diseases. *Trends Food Sci. Technol.* **108**, 164–176. 10.1016/j.tifs.2020.12.019 (2021).

[4] Cave, N. J. Hydrolyzed protein diets for dogs and cats. *Vet Clin North Am Small Anim Pract* 36, 1251–1268, vi; https://doi.org: (2006). 10.1016/j.cvsm.2006.08.008 10.1016/j.cvsm.2006.08.008 17085233

[5] Wei, Y. et al. The effect of dietary protein hydrolysate from black soldier fly larvae and *Schizochytrium* on palatability, nutrient metabolites and health status in beagle dogs. *Metabolites* **14**, 165. 10.3390/metabo14030165 (2024). 38535325 10.3390/metabo14030165 PMC10971824

[6] Hsu, C., Marx, F., Guldenpfennig, R., Valizadegan, N. & de Godoy, M. R. C. The effects of hydrolyzed protein on macronutrient digestibility, fecal metabolites and microbiota, oxidative stress and inflammatory biomarkers, and skin and coat quality in adult dogs. *J. Anim. Sci.* **102**, skae057. 10.1093/jas/skae057 (2024). 38442226 10.1093/jas/skae057 PMC10959486

[7] Strompfová, V. et al. Effect of hydrolyzed yeast administration on faecal microbiota, haematology, serum biochemistry and cellular immunity in healthy dogs. *Probiotics Antimicrob. Proteins*. **13**, 1267–1276. 10.1007/s12602-021-09765-9 (2021). 33710512 10.1007/s12602-021-09765-9

[8] Guilherme-Fernandes, J. et al. Squid meal and shrimp hydrolysate as novel protein sources for dog food. *Front. Vet. Sci.* **11**, 1360939. 10.3389/fvets.2024.1360939 (2024). 38450029 10.3389/fvets.2024.1360939 PMC10915000

[9] Zinn, K. E. et al. Fish protein substrates can substitute effectively for poultry by-product meal when incorporated in high-quality senior dog diets. *J. Anim. Physiol. Anim. Nutr.* **93**, 447–455. 10.1111/j.1439-0396.2008.00826.x (2009). 10.1111/j.1439-0396.2008.00826.x 18492029

[10] Pinto, C. F. D. et al. Hydrolyzed chicken liver used as single source of animal protein in diet and its effect on cytokines, immunoglobulins, and fecal microbiota profile of adult dogs. *PLoS One*. **17**, e0271932. 10.1371/journal.pone.0271932 (2022). 35867776 10.1371/journal.pone.0271932 PMC9307193

[11] Khan, A. I. et al. Shrimp peptide hydrolysate modulates the immune response in cyclophosphamide immunosuppressed mice model. *J. Food Biochem.* **46**, e14251. 10.1111/jfbc.14251 (2022). 35633198 10.1111/jfbc.14251

[12] Khan, A. I. et al. Effects of shrimp peptide hydrolysate on intestinal microbiota restoration and immune modulation in cyclophosphamide-treated mice. *Molecules* **27**, 1720. 10.3390/molecules27051720 (2022). 35268821 10.3390/molecules27051720 PMC8911659

[13] Hu, L. et al. Microbiome and metabolite analysis insight into the potential of shrimp head hydrolysate to alleviate depression-like behaviour in growth-period mice exposed to chronic stress. *Nutrients* **16**, 1953. 10.3390/nu16121953 (2024). 38931307 10.3390/nu16121953 PMC11206410

[14] Gunathilaka, B. E. et al. Evaluation of shrimp protein hydrolysate and Krill meal supplementation in low fish meal diet for red seabream (*Pagrus major*). *Fish. Aquat. Sci.* **24**, 109–120. 10.47853/FAS.2021.e11 (2021).

[15] Khosravi, S. et al. Effects of protein hydrolysates supplementation in low fish meal diets on growth performance, innate immunity and disease resistance of red sea Bream *Pagrus major*. *Fish. Shellfish Immunol.* **45**, 858–868. 10.1016/j.fsi.2015.05.039 (2015). 26074096 10.1016/j.fsi.2015.05.039

[16] Gisbert, E., Fournier, V., Solovyev, M., Skalli, A. & Andree, K. B. Diets containing shrimp protein hydrolysates provided protection to European sea bass (*Dicentrarchus labrax*) affected by a Vibrio pelagius natural infection outbreak. *Aquaculture* **495**, 136–143. 10.1016/j.aquaculture.2018.04.051 (2018).

[17] Lasekan, A. *Attenuating the Antibody Reactivity of the Shrimp Major Allergen (tropomyosin) Using Food Processing Methods* (University of Maine, 2017).

[18] Guilherme-Fernandes, J. et al. Unveiling the effects of shrimp hydrolysate as a dietary ingredient in healthy adult beagle dogs. *J. Anim. Sci.* **102**, skae280. 10.1093/jas/skae280 (2024). 39292957 10.1093/jas/skae280 PMC11484800

[19] Acuff, H. L., Dainton, A. N., Dhakal, J., Kiprotich, S. & Aldrich, G. Sustainability and pet food: Is there a role for veterinarians?? *Vet. Clin. N Am. : Small Anim. Pract.* **51**, 563–581. 10.1016/j.cvsm.2021.01.010 (2021). 10.1016/j.cvsm.2021.01.010 33773646

[20] Vasconcellos, R. S., Volpato, J. A. & Silva, I. C. Bioactive peptides extracted from hydrolyzed animal byproducts for dogs and cats. *Anim. Front.* **14**, 38–45. 10.1093/af/vfae012 (2024). 38910953 10.1093/af/vfae012 PMC11188960

[21] Kahn, C. M. *The Merck veterinary manual*. 9th edn, (2005).

[22] Kaneko, J. J., Harvey, J. W. & Bruss, M. L. *Clinical biochemistry of domestic animals*. 6th edn, (2008).

[23] Nothdurft, W. & Kreja, L. Hemopoietic progenitor cells in the blood as indicators of the functional status of the bone marrow after total-body and partial-body irradiation: Experiences from studies in dogs. *Stem Cells*. **16**, 97–111. 10.1002/stem.5530160813 (1998). 11012152 10.1002/stem.5530160813

[24] Matucci, A., Vultaggio, A., Maggi, E. & Kasujee, I. Is IgE or eosinophils the key player in allergic asthma pathogenesis? Are we asking the right question? *Respir Res.* **19**, 113. 10.1186/s12931-018-0813-0 (2018). 29879991 10.1186/s12931-018-0813-0 PMC5992661

[25] Wilkie, J. S., Yager, J. A., Eyre, P. & Parker, W. M. Morphometric analyses of the skin of dogs with atopic dermatitis and correlations with cutaneous and plasma Histamine and total serum IgE. *Vet. Pathol.* **27**, 179–186. 10.1177/030098589002700305 (1990). 2353419 10.1177/030098589002700305

[26] Sharkey, S. J. et al. A narrative review of the anti-hyperglycemic and satiating effects of fish protein hydrolysates and their bioactive peptides. *Mol. Nutr. Food Res.* **64**, 2000403. 10.1002/mnfr.202000403 (2020). 10.1002/mnfr.202000403 32939966

[27] Theysgeur, S. et al. New bioactive peptides identified from a tilapia byproduct hydrolysate exerting effects on DPP-IV activity and intestinal hormones regulation after canine Gastrointestinal simulated digestion. *Molecules* **26**, 136. 10.3390/molecules26010136 (2020). 33396793 10.3390/molecules26010136 PMC7796187

[28] Kim, J. H. et al. Short communication: pet foods with yeast hydrolysate can reduce body weight and increase girth in beagle dogs. *Can. J. Anim. Sci.* **92**, 207–210. 10.4141/cjas2011-123 (2012).

[29] Brownlee, M. The pathobiology of diabetic complications: A unifying mechanism. *Diabetes* **54**, 1615–1625. 10.2337/diabetes.54.6.1615 (2005). 15919781 10.2337/diabetes.54.6.1615

[30] Plecitá-Hlavatá, L. et al. Glucose-stimulated insulin secretion fundamentally requires H_2_O_2_ signaling by NADPH oxidase 4. *Diabetes* **69**, 1341–1354. 10.2337/db19-1130 (2020). 32245800 10.2337/db19-1130

[31] González, P., Lozano, P., Ros, G. & Solano, F. Hyperglycemia and oxidative stress: an integral, updated and critical overview of their metabolic interconnections. *Int. J. Mol. Sci.* **24**, 9352. 10.3390/ijms24119352 (2023). 37298303 10.3390/ijms24119352 PMC10253853

[32] Brennan, K. & Zheng, J. in *In xPharm: the Comprehensive Pharmacology Reference*. 1–4 (eds Enna, S. J., David, B. & Bylund) (Elsevier, 2007).

[33] Hassan, H. T. & Zander, A. Stem cell factor as a survival and growth factor in human normal and malignant hematopoiesis. *Acta Haematol.* **95**, 257–262. 10.1159/000203893 (1996). 8677752 10.1159/000203893

[34] Gooya, J. et al. Interleukin-8 directly synergizes with steel factor to promote the growth of lineage-negative c-kit-positive progenitors (abstract). *Exp. Hematol.* **24**, 1037 (1996).

[35] Laterveer, L., Lindley, I. J. D., Hamilton, M. S., Willemze, R. & Fibbe, W. E. Interleukin-8 induces rapid mobilization of hematopoietic stem cells with radioprotective capacity and long-term myelolymphoid repopulating ability. *Blood* **85**, 2269–2275. 10.1182/blood.V85.8.2269.bloodjournal8582269 (1995). 7718900

[36] Belpaire, A., van Geel, N., Speeckaert, R. & From IL-17 to IFN-γ in inflammatory skin disorders: is transdifferentiation a potential treatment target? *Front. Immunol.* **13**, 932265. 10.3389/fimmu.2022.932265 (2022). 35967358 10.3389/fimmu.2022.932265 PMC9367984

[37] Shao, H., Kaplan, H. J. & Sun, D. Bidirectional effect of IFN-γ on Th17 responses in experimental autoimmune uveitis. *Front. Ophthalmol.* **2**, 831084. 10.3389/fopht.2022.831084 (2022). 10.3389/fopht.2022.831084 PMC9521044 36188211

[38] Zhang, Q. et al. A high MCT-based ketogenic diet suppresses Th1 and Th17 responses to ameliorate experimental autoimmune encephalomyelitis in mice by inhibiting GSDMD and JAK2-STAT3/4 pathways. *Mol. Nutr. Food Res.* **68**, e2300602. 10.1002/mnfr.202300602 (2024). 38054637 10.1002/mnfr.202300602

[39] Pesce, B. et al. TNF-α affects signature cytokines of Th1 and Th17 T cell subsets through differential actions on TNFR1 and TNFR2. *Int. J. Mol. Sci.* **23**10.3390/ijms23169306 (2022). 10.3390/ijms23169306 PMC9408897 36012570

[40] Mitoma, H. et al. Infliximab induces potent anti-inflammatory responses by outside-to-inside signals through transmembrane TNF-α. *Gastroenterology* **128**, 376–392. 10.1053/j.gastro.2004.11.060 (2005). 15685549 10.1053/j.gastro.2004.11.060

[41] de Waal Malefyt, R., Abrams, J., Bennett, B., Figdor, C. G. & de Vries, J. E. Interleukin 10(IL-10) inhibits cytokine synthesis by human monocytes: An autoregulatory role of IL-10 produced by monocytes. *J. Exp. Med.* **174**, 1209–1220. 10.1084/jem.174.5.1209 (1991). 1940799 10.1084/jem.174.5.1209 PMC2119001

[42] Shouval, D. S. et al. *Chapter five - Interleukin 10 Receptor Signaling: Master Regulator of Intestinal Mucosal Homeostasis in Mice and Humans*Vol. 122 (Academic, 2014). 10.1016/B978-0-12-800267-4.00005-5 PMC4741283 24507158

[43] Teixeira, A. F. et al. Identification of leptospiral protein antigens recognized by WC1^+^ γδ T cell subsets as target for development of Recombinant vaccines. *Infect. Immun.* **90**, e0049221. 10.1128/iai.00492-21 (2022). 34694919 10.1128/IAI.00492-21 PMC8788707

[44] Novak, A. et al. Cellular and humoral immune responsiveness to inactivated *Leptospira interrogans* in dogs vaccinated with a tetravalent Leptospira vaccine. *Vaccine* **41**, 119–129. 10.1016/j.vaccine.2022.11.017 (2023). 36411135 10.1016/j.vaccine.2022.11.017

[45] Collins, N. Dietary regulation of memory T cells. *Int. J. Mol. Sci.* **21**, 4363. 10.3390/ijms21124363 (2020). 32575427 10.3390/ijms21124363 PMC7352243

[46] Masuda, K., Sato, A., Tanaka, A. & Kumagai, A. Hydrolyzed diets May stimulate food-reactive lymphocytes in dogs. *J. Vet. Med. Sci.* **82**, 177–183. 10.1292/jvms.19-0222 (2020). 31875597 10.1292/jvms.19-0222 PMC7041975

[47] He, X. Q., Cao, W. H., Pan, G. K., Yang, L. & Zhang, C. H. Enzymatic hydrolysis optimization of *Paphia undulata* and lymphocyte proliferation activity of the isolated peptide fractions. *J. Sci. Food Agric.* **95**, 1544–1553. 10.1002/jsfa.6859 (2015). 25087732 10.1002/jsfa.6859

[48] Hou, H., Fan, Y., Li, B., Xue, C. & Yu, G. Preparation of Immunomodulatory hydrolysates from Alaska Pollock frame. *J. Sci. Food Agric.* **92**, 3029–3038. 10.1002/jsfa.5719 (2012). 22576701 10.1002/jsfa.5719

[49] Wang, Y. K. et al. Oyster (*Crassostrea gigas*) hydrolysates produced on a plant scale have antitumor activity and immunostimulating effects in balb/c mice. *Mar. Drugs*. **8**, 255–268. 10.3390/md8020255 (2010). 20390104 10.3390/md8020255 PMC2852837

[50] Cai, B., Pan, J., Wu, Y., Wan, P. & Sun, H. Immune functional impacts of oyster peptide-based enteral nutrition formula (OPENF) on mice: A pilot study. *Chin. J. Oceanol. Limnol.* **31**, 813–820. 10.1007/s00343-013-2311-z (2013).

[51] Zannoni, A. et al. Non-invasive assessment of fecal stress biomarkers in hunting dogs during exercise and at rest. *Front. Vet. Sci.* **7**10.3389/fvets.2020.00126 (2020). 10.3389/fvets.2020.00126 PMC7186473 32373631

[52] Vykoukal, D. & Davies, M. G. Vascular biology of metabolic syndrome. *J. Vasc Surg.* **54**, 819–831. 10.1016/j.jvs.2011.01.003 (2011). 21439758 10.1016/j.jvs.2011.01.003 PMC3136643

[53] Wu, S., Dong, K., Wang, J. & Bi, Y. Tumor necrosis factor alpha improves glucose homeostasis in diabetic mice independent with tumor necrosis factor receptor 1 and tumor necrosis factor receptor 2. *Endocr. J.* **65**, 601–609. 10.1507/endocrj.EJ17-0539 (2018). 29576600 10.1507/endocrj.EJ17-0539

[54] Arroyo Hornero, R., Hamad, I., Côrte-Real, B. & Kleinewietfeld, M. The impact of dietary components on regulatory T cells and disease. *Front. Immunol.* **11**, 00253. 10.3389/fimmu.2020.00253 (2020). 10.3389/fimmu.2020.00253 PMC7047770 32153577

[55] Alhalwani, A. Y., Abudawood, K., Qadizadah, A. B. E. A., Jambi, S. & Sannan, N. S. Immunoglobulin A levels and its correlation with neutrophil-to-lymphocyte ratio as inflammatory biomarkers for dry eye disease in type 2 diabetes: a retrospective study. *Front. Immunol.* **14**, 1184862. 10.3389/fimmu.2023.1184862 (2023). 37520541 10.3389/fimmu.2023.1184862 PMC10375287

[56] Hiney, K. et al. Fecal microbiota composition, serum metabolomics, and markers of inflammation in dogs fed a Raw meat-based diet compared to those on a kibble diet. *Front. Vet. Sci.* **11**10.3389/fvets.2024.1328513 (2024). 10.3389/fvets.2024.1328513 PMC11061498 38694479

[57] Maria, A. P. J. et al. The effect of age and carbohydrate and protein sources on digestibility, fecal microbiota, fermentation products, fecal iga, and immunological blood parameters in dogs. *J. Anim. Sci.* **95**, 2452–2466. 10.2527/jas.2016.1302 (2017). 28727033 10.2527/jas.2016.1302

[58] Wei, Y. et al. Influence of fish protein hydrolysate on intestinal health and microbial communities in turbot Scophthalmus maximus. *Aquaculture* **576**, 739827. 10.1016/j.aquaculture.2023.739827 (2023).

[59] Duarte, J., Vinderola, G., Ritz, B., Perdigón, G. & Matar, C. Immunomodulating capacity of commercial fish protein hydrolysate for diet supplementation. *Immunobiology* **211**, 341–350. 10.1016/j.imbio.2005.12.002 (2006). 16716803 10.1016/j.imbio.2005.12.002

[60] Takeuchi, T. & Ohno, H. IgA in human health and diseases: Potential regulator of commensal microbiota. *Front. Immunol.* **13**10.3389/fimmu.2022.1024330 (2022). 36439192 10.3389/fimmu.2022.1024330 PMC9685418

[61] Hapfelmeier, S. et al. Reversible microbial colonization of germ-free mice reveals the dynamics of IgA immune responses. *Science* **328**, 1705–1709. 10.1126/science.1188454 (2010). 20576892 10.1126/science.1188454 PMC3923373

[62] Soontararak, S. et al. Humoral immune responses against gut bacteria in dogs with inflammatory bowel disease. *PLoS One*. **14**, e0220522. 10.1371/journal.pone.0220522 (2019). 31369623 10.1371/journal.pone.0220522 PMC6675102

[63] Deng, P. & Swanson, K. S. Gut microbiota of humans, dogs and cats: Current knowledge and future opportunities and challenges. *Br. J. Nutr.* **113**, S6–S17. 10.1017/S0007114514002943 (2015). 25414978 10.1017/S0007114514002943

[64] Pereira, A. M. et al. Effects of zinc source and enzyme addition on the fecal microbiota of dogs. *Front. Microbiol.* **12**, 688392. 10.3389/fmicb.2021.688392 (2021). 34721312 10.3389/fmicb.2021.688392 PMC8549731

[65] Yang, J. et al. Oscillospira - a candidate for the next-generation probiotics. *Gut Microbes*. **13**, 1987783. 10.1080/19490976.2021.1987783 (2021). 34693878 10.1080/19490976.2021.1987783 PMC8547878

[66] Tremaroli, V. & Bäckhed, F. Functional interactions between the gut microbiota and host metabolism. *Nature* **489**, 242–249. 10.1038/nature11552 (2012). 22972297 10.1038/nature11552

[67] Sridharan, G. V. et al. Prediction and quantification of bioactive microbiota metabolites in the mouse gut. *Nat. Commun.* **5**, 5492. 10.1038/ncomms6492 (2014). 25411059 10.1038/ncomms6492

[68] Silva, Y. P., Bernardi, A. & Frozza, R. L. The role of short-chain fatty acids from gut microbiota in gut-brain communication. *Front. Endocrinol.* **11**, 25. 10.3389/fendo.2020.00025 (2020). 10.3389/fendo.2020.00025 PMC7005631 32082260

[69] Ecklu-Mensah, G. et al. Gut microbiota and fecal short chain fatty acids differ with adiposity and country of origin: The METS-microbiome study. *Nat. Commun.* **14**, 5160. 10.1038/s41467-023-40874-x (2023). 37620311 10.1038/s41467-023-40874-x PMC10449869

[70] Mentula, S. et al. Comparison between cultured small-intestinal and fecal microbiotas in beagle dogs. *Appl. Environ. Microbiol.* **71**, 4169–4175. 10.1128/AEM.71.8.4169-4175.2005 (2005). 16085799 10.1128/AEM.71.8.4169-4175.2005 PMC1183360

[71] Bermingham, E. N., Maclean, P., Thomas, D. G., Cave, N. J. & Young, W. Key bacterial families (Clostridiaceae, Erysipelotrichaceae and Bacteroidaceae) are related to the digestion of protein and energy in dogs. *PeerJ* **5**, e3019. 10.7717/peerj.3019 (2017). 28265505 10.7717/peerj.3019 PMC5337088

[72] Lin, R., Liu, W., Piao, M. & Zhu, H. A review of the relationship between the gut microbiota and amino acid metabolism. *Amino Acids*. **49**, 2083–2090. 10.1007/s00726-017-2493-3 (2017). 28932911 10.1007/s00726-017-2493-3

[73] Vestergaard, M. V. et al. Gut microbiota signatures in inflammatory bowel disease. *United Eur. Gastroenterol. J.* **12**, 22–33. 10.1002/ueg2.12485 (2024). 10.1002/ueg2.12485 PMC10859715 38041519

[74] Díaz-Regañón, D. et al. Characterization of the fecal and mucosa-associated microbiota in dogs with chronic inflammatory enteropathy. *Animals* **13**, 326. 10.3390/ani13030326 (2023). 36766216 10.3390/ani13030326 PMC9913788

[75] Laflamme, D. Development and validation of a body condition score system for dogs. *Canine Pract.* **22**, 10–15 (1997).

[76] FEDIAF. *Nutritional Guidelines for Complete and Complementary Pet Food for Cats and Dogs* (Bruxelles, 2021).

[77] Leduc, A. et al. Shrimp by-product hydrolysate induces intestinal myotropic activity in European Seabass (*Dicentrarchus labrax*). *Aquaculture* **497**, 380–388. 10.1016/j.aquaculture.2018.08.009 (2018).

[78] Segeritz, C. P. & Vallier, L. in *Basic Science Methods for Clinical Researchers* (eds Morteza Jalali, Francesca Y. L. Saldanha, & Mehdi Jalali) 151–172Academic Press, (2017).

[79] Correia, A. et al. Mucosal and systemic T cell response in mice intragastrically infected with *Neospora Caninum* tachyzoites. *Vet. Res.* **44**, 69. 10.1186/1297-9716-44-69 (2013). 23937079 10.1186/1297-9716-44-69 PMC3751650

[80] Moreira, M. L. et al. Cross-reactivity of commercially available anti-human monoclonal antibodies with canine cytokines: establishment of a reliable panel to detect the functional profile of peripheral blood lymphocytes by intracytoplasmic staining. *Acta Vet. Scand.* **57**, 51. 10.1186/s13028-015-0142-y (2015). 26362860 10.1186/s13028-015-0142-y PMC4566394

[81] Peters, I. R., Calvert, E. L., Hall, E. J. & Day, M. J. Measurement of Immunoglobulin concentrations in the feces of healthy dogs. *Clin. Diagn. Lab. Immunol.* **11**, 841–848. 10.1128/cdli.11.5.841-848.2004 (2004). 15358641 10.1128/CDLI.11.5.841-848.2004 PMC515266

[82] Walters, W. et al. Improved bacterial 16S rRNA gene (V4 and V4-5) and fungal internal transcribed spacer marker gene primers for microbial community surveys. *mSystems* 1 (2016). 10.1128/mSystems.00009-15 10.1128/mSystems.00009-15 PMC5069754 27822518

[83] Bolyen, E. et al. Reproducible, interactive, scalable and extensible Microbiome data science using QIIME 2. *Nat. Biotechnol.* **37**, 852–857. 10.1038/s41587-019-0209-9 (2019). 31341288 10.1038/s41587-019-0209-9 PMC7015180

[84] Martin, M. Cutadapt removes adapter sequences from high-throughput sequencing reads. *EMBnet J.* **17**, 3. 10.14806/ej.17.1.200 (2011).

[85] Callahan, B. J. et al. DADA2: High-resolution sample inference from illumina amplicon data. *Nat. Methods*. **13**, 581–583. 10.1038/nmeth.3869 (2016). 27214047 10.1038/nmeth.3869 PMC4927377

[86] Rognes, T., Flouri, T., Nichols, B., Quince, C. & Mahé, F. VSEARCH: A versatile open source tool for metagenomics. *PeerJ* **4**, e2584. 10.7717/peerj.2584 (2016). 27781170 10.7717/peerj.2584 PMC5075697

[87] Pedregosa, F. et al. Scikit-learn: machine learning in Python. *J. Mach. Learn. Res.* **12**, 2825–2830 (2012).

[88] Quast, C. et al. The SILVA ribosomal RNA gene database project: improved data processing and web-based tools. *Nucleic Acids Res.* **41**, D590–596. 10.1093/nar/gks1219 (2013). 23193283 10.1093/nar/gks1219 PMC3531112

[89] Robeson, M. S. RESCRIPt: reproducible sequence taxonomy reference database management. *PLoS Comp. Biol.* **17**, e1009581. 10.1371/journal.pcbi.1009581 (2021). 10.1371/journal.pcbi.1009581 PMC8601625 34748542

[90] Shannon, C. E. A mathematical theory of communication. *Bell Syst. Tech. J.* **27**, 379–423. 10.1002/j.1538-7305.1948.tb01338.x (1948).

[91] Faith, D. P. Conservation evaluation and phylogenetic diversity. *Biol. Conserv.* **61**, 1–10. 10.1016/0006-3207(92)91201-3 (1992).

[92] Fisher, R. A., Corbet, A. S. & Williams, C. B. The relation between the number of species and the number of individuals in a random sample of an animal population. *J. Anim. Ecol.* **12**, 42–58. 10.2307/1411 (1943).

[93] Martino, C. et al. Context-aware dimensionality reduction deconvolutes gut microbial community dynamics. *Nat. Biotechnol.* **39**, 165–168. 10.1038/s41587-020-0660-7 (2021). 32868914 10.1038/s41587-020-0660-7 PMC7878194

[94] Bray, J. R. & Curtis, J. T. An ordination of the upland forest communities of Southern Wisconsin. *Ecol. Monogr.* **27**, 325–349. 10.2307/1942268 (1957).

[95] Fedarko, M. W. et al. Visualizing ‘omic feature rankings and log-ratios using Qurro. *NAR Genom Bioinform*. **2**, lqaa023. 10.1093/nargab/lqaa023 (2020). 32391521 10.1093/nargab/lqaa023 PMC7194218

[96] Anderson, M. J. in *Wiley StatsRef: Statistics Reference Online* 1–15.

[97] Seabold, S. & Perktold, J. Statsmodels: econometric and statistical modeling with Python. *Proceedings of the 9th Python in Science Conference* (2010). (2010).

[98] Lin, H. & Peddada, S. D. Analysis of compositions of microbiomes with bias correction. *Nat. Commun.* **11**, 3514. 10.1038/s41467-020-17041-7 (2020). 32665548 10.1038/s41467-020-17041-7 PMC7360769

