# Supplementary material for: Unraveling the role of shrimp hydrolysate as a food supplement in the immune function and fecal microbiota of beagle dogs
Source: Sci Rep. 2025 Jul 15;15:25510. doi: 10.1038/s41598-025-09942-8 (PMC12263991; doi:10.1038/s41598-025-09942-8)
Supplement: Supplementary file 1 — Supplementary Material 1 [file 41598_2025_9942_MOESM1_ESM.docx]

Table S1. Hematology, serum chemistry, C-reactive protein, and plasma immunoglobulin E in dogs fed the control and experimental diets over time.

| Item | Diet^1^ | | SEM^2^ | Week | | | SEM^2^ | *P* – value | | |
| --- | --- | --- | --- | --- | --- | --- | --- | --- | --- | --- |
|  | Control | Experimental |  | 4 | 8 | 12 |  | Diet | Week | Diet*Week |
| White blood cells, × 10^3^/μL | 6.71 | 7.67 | 0.291 | 6.97 | 7.23 | 7.37 | 0.344 | 0.002 | 0.601 | 0.139 |
| Neutrophils, % | 53.2 | 56.8 | 1.46 | 54.7 | 53.5 | 56.8 | 1.52 | 0.036 | 0.129 | 0.168 |
| Lymphocytes, % | 35.7 | 33.1 | 1.54 | 35.4 | 34.9 | 32.8 | 1.53 | 0.183 | 0.265 | 0.219 |
| Monocytes, % | 5.58 | 5.31 | 0.321 | 5.23 | 5.32 | 5.80 | 0.362 | 0.437 | 0.347 | 0.734 |
| Eosinophils, % | 5.51 | 4.50 | 0.323 | 4.53^a^ | 6.07^b^ | 4.43^a^ | 0.372 | 0.017 | 0.003 | 0.671 |
| Red blood cells, × 10^6^/μL | 7.34 | 7.29 | 0.135 | 7.20 | 7.40 | 7.33 | 0.133 | 0.636 | 0.068 | 0.044 |
| Haemoglobin, g/dL | 17.3 | 17.1 | 0.332 | 16.9^a^ | 17.5^b^ | 17.3^a,b^ | 0.323 | 0.596 | 0.013 | 0.037 |
| Platelets, × 10^3^/μL | 274 | 300 | 12.4 | 309^b^ | 273^a^ | 280^a^ | 13.7 | 0.038 | 0.040 | 0.632 |
| Mean platelet volume, fL | 9.88 | 9.90 | 0.177 | 10.1^c^ | 9.88^b^ | 9.70^a^ | 0.162 | 0.914 | 0.004 | 0.650 |
| Total protein, g/dL | 5.71 | 5.86 | 0.066 | 5.56^a^ | 5.96^b^ | 5.84^a,b^ | 0.072 | 0.106 | 0.001 | 0.740 |
| Albumin, g/dL | 3.74 | 3.78 | 0.046 | 3.61^a^ | 3.85^b^ | 3.83^b^ | 0.042 | 0.565 | <0.001 | 0.657 |
| Globulin, g/dL | 1.98 | 2.07 | 0.034 | 1.95 | 2.11 | 2.01 | 0.043 | 0.067 | 0.054 | 0.720 |
| Glucose, mg/dL | 92.8 | 86.4 | 1.84 | 84.9^a^ | 98.1^b^ | 85.8^a^ | 1.83 | 0.023 | <0.001 | 0.608 |
| Creatinine, mg/dL | 0.802 | 0.812 | 0.0536 | 0.800 | 0.837 | 0.785 | 0.0446 | 0.894 | 0.456 | 0.143 |
| Urea, mg/dL | 33.0 | 28.5 | 2.42 | 27.6 | 33.0 | 31.7 | 2.74 | 0.098 | 0.229 | 0.172 |
| Alanine aminotransferase, U/L | 28.4 | 31.8 | 2.20 | 32.1 | 28.3 | 29.9 | 2.50 | 0.218 | 0.471 | 0.273 |
| Alkaline phosphatase, U/L | 43.1 | 45.2 | 1.71 | 44.6 | 44.6 | 43.1 | 1.53 | 0.396 | 0.618 | 0.966 |
| C-reactive protein, μg/mL | 6.99 | 8.78 | 0.853 | 7.80 | 8.75 | 7.10 | 0.758 | 0.234 | 0.318 | 0.277 |
| Immunoglobulin E, μg/mL | 171 | 148 | 21.0 | 206^b^ | 209^b^ | 63.6^a^ | 18.5 | 0.438 | <0.001 | 0.135 |
| ^1^Control: complete diet without the inclusion of shrimp hydrolysate; Experimental: control diet with 5% of shrimp hydrolysate in replacement of wheat gluten.  ^2^SEM: Standard error of the mean.  ^a,b^Means with different lowercase superscript letters in the same row are significantly different (*P* < 0.05). | | | | | | | | | | |

Table S2. Concentration of cytokines, chemokine, and growth factors in serum of dogs fed the control and experimental diets over time.

| Item | Diet^1^ | | SEM^2^ | Week | | | SEM^2^ | *P* – value | | |  |
| --- | --- | --- | --- | --- | --- | --- | --- | --- | --- | --- | --- |
|  | Control | Experimental |  | 4 | 8 | 12 |  | Diet | Week | Diet*Week | |
| Il-12/IL-23p40, pg/mL | 580 | 1418 | 352.6 | 938 | 986 | 1072 | 253.4 | 0.105 | 0.239 | 0.114 | |
| IL-8, pg/mL | 1112 | 437 | 239.6 | 778 | 708 | 837 | 180.6 | 0.057 | 0.501 | 0.038 | |
| IL-2, pg/mL | 43.2 | 99.1 | 38.79 | 62.1 | 71.6 | 79.7 | 40.31 | 0.161 | 0.901 | 0.899 | |
| SCF, pg/mL | 27.3 | 54.9 | 14.44 | 36.7 | 40.5 | 46.1 | 10.52 | 0.188 | 0.219 | 0.026 | |
| MCP-1, pg/mL | 103 | 100 | 12.3 | 104 | 106 | 94.3 | 9.74 | 0.846 | 0.281 | 0.379 | |
| VEGF-A, pg/mL | 5.97 | 6.16 | 0.903 | 5.81 | 5.27 | 7.10 | 0.770 | 0.884 | 0.058 | 0.102 | |
| ^1^Control: complete diet without the inclusion of shrimp hydrolysate; Experimental: control diet with 5% of shrimp hydrolysate in replacement of wheat gluten.  ^2^SEM: Standard error of the mean.  Abbreviations: IL, interleukin; MCP-1, monocyte chemoattractant protein-1; SCF, stem cell factor; VEGF-A, vascular endothelial growth factor A. | | | | | | | | | | |  |

Table S3. Fold increase of total reactive oxygen species (ROS) and superoxide production in cells stimulated with phorbol myristate acetate (PMA) for 30 and 60 min over the basal (non-stimulated) in weeks 4, 8 and 12 in dogs fed the control and experimental diets.

| Item | Week 4 | |  | Week 8 | |  | Week 12 | | SEM^2^ | *P* - value | | |
| --- | --- | --- | --- | --- | --- | --- | --- | --- | --- | --- | --- | --- |
|  | Control^1^ | Experimental^1^ | | Control^1^ | Experimental^1^ | | Control^1^ | Experimental^1^ |  | Diet | Week | Diet*Week |
| Total ROS production |  |  |  |  |  |  |  |  |  |  |  |  |
| 30 min PMA stimulation | 6.2 | 1.85 |  | 1.02 | 0.785 |  | 43.7 | 42.7 | 5.638 | 0.642 | <0.001 | 0.930 |
| 60 min PMA stimulation | 2.58^a^ | 1.92^a^ |  | 1.51^a^ | 0.800^a^ |  | 13.2^b^ | 18.0^b^ | 1.176 | 0.174 | <0.001 | 0.030 |
| Superoxide production |  |  |  |  |  |  |  |  |  |  |  |  |
| 30 min PMA stimulation | 1.82 | 1.28 |  | 2.26 | 1.9 |  | 10.4 | 3.94 | 1.523 | 0.066 | 0.002 | 0.088 |
| 60 min PMA stimulation | 2.86^a^ | 1.23^a^ |  | 4.32^a^ | 3.04^a^ |  | 10.7^b^ | 3.79^a^ | 1.144 | 0.002 | <0.001 | 0.038 |
| ^1^Control: complete diet without the inclusion of shrimp hydrolysate; Experimental: control diet with 5% of shrimp hydrolysate in replacement of wheat gluten. ^2^SEM: Standard error of the mean. ^a,b^Means with different lowercase superscript letters in the same row are significantly different (*P* < 0.05). | | | | | | | | | | | |  |

Table S4. Concentration of cytokines in the supernatants of PBMC stimulated with concanavalin A in dogs fed the control and experimental diets over time.

| Item | Diet^1^ | | SEM^2^ | Week | | | SEM^2^ | *P* - value | | |
| --- | --- | --- | --- | --- | --- | --- | --- | --- | --- | --- |
|  | Control | Experimental |  | 4 | 8 | 12 |  | Diet | Week | Diet*Week |
| IL-17, pg/mL | 1310 | 986 | 306.8 | 402^a^ | 1243^a,b^ | 1800^b^ | 374.4 | 0.405 | 0.027 | 0.752 |
| IFN-γ, pg/mL | 1885 | 1461 | 384.1 | 2194^b^ | 1735^a,b^ | 1092^a^ | 328.6 | 0.452 | 0.011 | 0.122 |
| TNF-α, pg/mL | 30.6 | 28.2 | 3.31 | 27.3^a^ | 19.2^a^ | 41.6^b^ | 4.23 | 0.594 | 0.004 | 0.317 |
| IL-10, pg/mL | 193 | 144 | 28.1 | 104^a^ | 152^a^ | 250^b^ | 33.7 | 0.124 | 0.002 | 0.729 |

^1^Control: complete diet without inclusion of shrimp hydrolysate; Experimental: control diet with 5% of shrimp hydrolysate in replacement of 5% of wheat gluten.

^2^SEM: Standard error of the mean.

^a,b^Means with different lowercase superscript letters in the same row are significantly different (*P* < 0.05).

Abbreviations: IFN-γ: interferon-gamma; IL, interleukin; TNF-α: tumor necrosis factor alpha.

Table S5. Percentage of CD3^+^CD4^+^ and CD3^+^CD8^+^ cells expressing interferon-gamma, tumor necrosis factor alpha and both cytokines, and the CD4^+^/CD8^+^ ratio in weeks 4, 8, and 12.

| Item | Week | | | SEM^1^ | *P* - value | | |
| --- | --- | --- | --- | --- | --- | --- | --- |
|  | 4 | 8 | 12 |  | Diet | Week | Diet*Week |
| CD3^+^CD4^+^, % | 63.9^b^ | 59.6^a^ | 59.0^a^ | 1.46 | 0.190 | 0.050 | 0.585 |
| CD4^+^TNFα^+^IFNγ^+^, % | 8.76^a^ | 14.1^b^ | 15.2^b^ | 1.413 | 0.113 | 0.002 | 0.290 |
| CD4^+^TNFα^+^IFNγ^-^, % | 13.4^a^ | 16.2^a^ | 20.3^b^ | 1.07 | <0.001 | <0.001 | 0.607 |
| CD4^+^TNFα^-^IFNγ^+^, % | 5.92^a^ | 8.62^c^ | 7.26^b^ | 1.041 | 0.748 | 0.023 | 0.927 |
| CD3^+^CD8^+^, % | 13.6 | 13.8 | 14.6 | 0.59 | 0.180 | 0.491 | 0.187 |
| CD8^+^TNFα^+^IFNγ^+^, % | 8.69^a^ | 27.6^b^ | 32.7^b^ | 4.534 | 0.251 | <0.001 | 0.832 |
| CD8^+^TNFα^+^IFNγ^-^, % | 4.41 | 6.32 | 5.58 | 0.586 | <0.001 | 0.054 | 0.890 |
| CD8^+^TNFα^-^IFNγ^+^, % | 7.73^a^ | 17.4^b^ | 16.9^b^ | 2.669 | 0.216 | <0.001 | 0.526 |
| CD4^+^/CD8^+^ | 5.23 | 4.56 | 4.26 | 0.385 | 0.951 | 0.317 | 0.354 |

^1^SEM: Standard error of the mean.

^a,b,c^Means with different lowercase superscript letters in the same row are significantly different (*P* < 0.05).

Abbreviations: IFN-γ: interferon-gamma; TNF-α: tumor necrosis factor alpha.

Table S6. Chemical composition of the control and experimental diets.

| Item | Diet^1^ | |
| --- | --- | --- |
|  | Control | Experimental |
| Dry matter, DM, g/kg | 942 | 957 |
| Ash, g/kg DM | 77.7 | 79.9 |
| Crude protein, g/kg DM | 293 | 293 |
| Ether extract, g/kg DM | 136 | 131 |
| Neutral detergent fiber, g/kg DM | 146 | 143 |
| Starch, g/kg DM | 330 | 319 |
| Gross energy, MJ/kg DM | 19.2 | 19.2 |
| Data reported in Guilherme-Fernandes et al. (2024).  ^1^Complete diets included, in descending order, poultry by-product meal, corn, wheat, broken rice, pea starch, wheat gluten, poultry and mammal fat, pea protein concentrate, autolyzed brewers' yeast, beet pulp, lucerne, fish oil; Control diet: complete diet without the inclusion of shrimp hydrolysate; Experimental diet: control diet with 5% of shrimp hydrolysate in replacement of wheat gluten. | | |

Figure S1. Representative example of flow cytometry gating strategy used to define the production of total reactive oxygen species (ROS) and superoxide in non-stimulated cells (basal ROS production) and in cells stimulated with phorbol myristate acetate for 30 and 60 min. Cells were gated based on side scatter (SSC) and forward scatter (FSC) parameters and doublets were excluded (Single cells) in FSC versus FSC plots. Mean fluorescence intensity was used to quantify total ROS and superoxide production.

Figure S2. Representative example of flow cytometry gating strategy used to define lymphocyte proliferation of non-stimulated CD3^+^CD4^+^ cells or in response to recombinant antigen from *Leptospira interrogans* and concanavalin A. Lymphocytes were gated based on side scatter (SSC) and forward scatter (FSC) parameters and doublets were excluded (Single cells) in FSC versus FSC plots. Viable cells (Live cells) were defined as propidium iodide (PI) negative cells. CD3^+^CD8^+^ cells and CD3^+^CD4^+^ cells were set in contour plots, based on fluorescence minus one (FMO) staining.

Figure S3. Representative example of flow cytometry gating strategy used to define lymphocyte intracellular cytokines tumor necrosis factor-alpha (TNF-α) and interferon-gamma (IFN-γ). Lymphocytes were gated based on side scatter (SSC) and forward scatter (FSC) parameters and doublets were excluded (Single cells) in FSC versus FSC plots. Viable cells (Live cells) were defined as Fixable Viability Dye (FVD) negative cells. CD3^+^CD8^+^ cells and CD3^+^CD4^+^ cells expressing TNF-α and/or IFN-γ were set in contour plots, based on fluorescence minus one (FMO) staining.

Figure S4. Representative example of flow cytometry gating strategy used to define lymphocyte intracellular cytokine Foxp3. Lymphocytes were gated based on side scatter (SSC) and forward scatter (FSC) parameters and doublets were excluded (Single cells) in FSC versus FSC plots. Viable cells (Live cells) were defined as Fixable Viability Dye (FVD) negative cells. CD4^+^CD25^+^ expressing Foxp3 were set in contour plots, based on fluorescence minus one (FMO) staining.
